# Value of blood neural cell-derived small extracellular vesicles in the diagnosis and prediction of Alzheimer's disease: A systematic review

**DOI:** 10.1016/j.tjpad.2025.100193

**Published:** 2025-05-01

**Authors:** Weibing Pan, Yu Teng, Xiaowan Han, Shaojiao Liu, Xingxue Pang, Lei Wang, Mingjing Zhao

**Affiliations:** Dongzhimen Hospital, Beijing University of Chinese Medicine, Beijing, China

**Keywords:** Blood neural cell-derived small extracellular vesicles, Alzheimer's disease, Biomarker, Diagnosis, Prediction

## Abstract

Blood neural cell-derived small extracellular vesicles (sEVs) can directly reflect changes in brain tissue and are easier to obtain than cerebrospinal fluid. This article systematically reviews the alterations of proteins and miRNAs from neural cell-derived sEVs in patients with Alzheimer's disease (AD), and summarizes the biomarkers with clinical diagnostic and predictive value. PubMed, Web of Science, Embase, and Cochrane Library were searched for studies in blood neural cell-derived sEVs in AD patients up to May 2024. According to the inclusion and exclusion criteria, the literature was screened, the information was extracted and the quality was evaluated. Proteins and miRNAs from neural cell-derived sEVs were classified and summarized, focusing on target molecules with high diagnostic and predictive values for AD. A final 34 articles reporting 5601 participants were included. In cross-sectional studies, Aβ- and Tau-related proteins (Aβ42, Aβ42/40, p-S396-Tau, p-Tau181), p-S312-IRS-1, and cathepsin D were increased, conversely, synaptic proteins (neurogranin, synaptotagmin, synaptophysin, synaptopodin, NMDAR2A) and REST were decreased in blood neuron-derived sEVs (NDsEVs) of patients with AD. While miR-29c-3p was increased in blood NDsEVs and glial cell-derived sEVs. Each of these proteins and miRNAs demonstrated high AD diagnostic value. Additionally, blood astrocyte-derived sEVs (ADsEVs) showed increased complement effector proteins and decreased complement regulatory proteins with a moderate diagnostic value. In longitudinal cohort studies, three composite models displayed high predictive efficacy for early AD prediction, and could predict the occurrence of AD within 1–10 years. Therefore, Aβ- and Tau-related proteins, synaptic proteins, and miRNA in blood neural cell-derived sEVs demonstrate high AD diagnostic and predictive values serving as important biomarkers. Especially, synaptic proteins showed significant changes in the early clinical stage, which has early predictive value.

## Highlights

• Blood neural cell-derived sEV Aβ- and tau-related proteins, synaptic proteins, and miRNAs are important biomarkers of AD.
• Blood neural cell-derived sEVs can predict the development of AD within 1–10 years.
• Compared with the existing blood diagnostic markers, the synaptic proteins in blood neural cell-derived sEVs show significant changes in the early clinical stage, which has the value of early diagnosis.
• Blood neural cell-derived sEVs, which are consistent with the changes of brain tissue, can not only detect pathological products related to AD, but also detect the changes of neurosynaptic proteins, and can better reflect the pathological changes of neurons than blood and CSF.
• Blood neural cell-derived sEVs have important clinical value for the diagnosis and early prediction of AD.

## Graphical abstract

**Figure:**
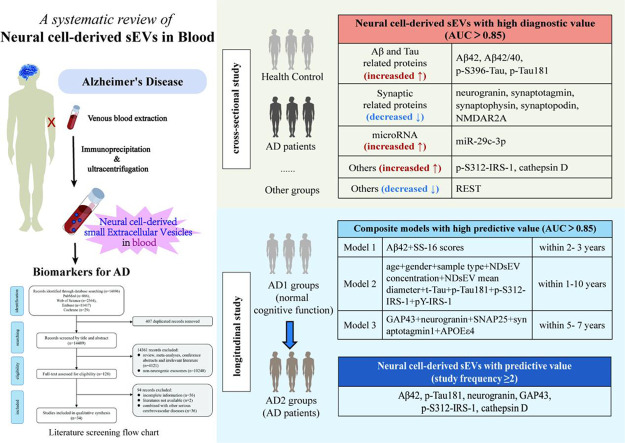

## 1 Introduction

Alzheimer's disease (AD) is a neurodegenerative disorder characterized by a progressive decline in cognitive abilities among older adults, significantly impacting their daily lives. According to the World Alzheimer Report 2023, the global prevalence of AD is projected to increase from 55 million in 2019 to 139 million by 2050 [1]. A 2019 study reported in the Journal of the American Medical Association found that there were 5.8 million patients with AD in the United States [2]. An epidemiological survey published in Lancet Neurology showed that the number of patients with AD in China had exceeded 10 million as of 2020, accounting for approximately one-fourth of the global patient population [3]. AD onset is occult, with mainly mild cognitive impairment (MCI) in the early stage, which is often ignored and has a long latent period. By diagnosis, the disease has already entered an irreversible stage. The onset of AD is hidden, with a long incubation period. At the initial stage, mild cognitive impairment (MCI) is the main symptom, and it has entered an irreversible stage when it is diagnosed later. It takes about 17 years for AD to develop from preclinical to the presence of typical evidence, such as cerebrospinal fluid (CSF) p-Tau and Aβ [4]. Current AD treatments can only partially improve symptoms and cannot reverse the pathological process. Therefore, identifying biomarkers for early AD diagnosis and prediction is of significant value.

According to the National Institute on Aging-Alzheimer's Association (NIA-AA) guidelines, the pathogenesis of Alzheimer's disease (AD) primarily involve "A/T/N/I" [[5], [6], [7]]. These include core biomarkers such as amyloid-beta (Aβ) and phosphorylated tau protein, as well as non-specific markers like total tau (t-Tau) and glial fibrillary acidic protein (GFAP). However, the invasive nature of CSF examination and the high cost of positron emission tomography (PET) scans render these methods unsuitable for routine examinations, limiting their feasibility for early detection. Related scales can also be used for auxiliary AD diagnosis, including the Mini Mental State Examination (MMSE), Clinical Dementia Rating Scale (CDR) and so on. However, these subjective scales have numerous interfering factors, rendering them unsuitable as a standard for accurate AD diagnosis. Therefore, there is an urgent clinical need for a convenient biomarker that does not cause trauma and is highly accuracy and sensitivity.

Liquid biopsy is a widely favored clinical detection technique, which aims to achieve diagnostic goals through the collection and analysis of fluids (e.g., blood, CSF, urine, saliva). Blood, the most widely used bodily fluid, holds significant clinical value in disease diagnosis [8,9]. Recent studies have shown that p-Tau217 is an emerging blood biomarker with significant diagnostic value [10,11]. However, peripheral blood primarily reflects systemic changes and cannot directly reflect the characteristics of the nervous system or neuronal specificity. Meanwhile, neural exosomes may offer a more direct source of biomarkers related to the nervous system. Recent studies have found that presynaptic failure is a strong physiopathological component of AD [12,13]. Several studies have demonstrated that the levels of synaptic proteins such as complexin-1, complexin-2, synaptotagmin-1, synaptogyrin-1, and syntaxin-1A are decreased in the brain tissues of patients with AD [14,15]. Given the inability to obtain brain tissue from patients, it is particularly important to find proteins in blood that can reflect synaptic-related functions for early prediction in the preclinical stage of AD. According to the Minimum Information for Studies of Extracellular Vesicles (MISEV 2023), small extracellular vesicles (sEVs) (mainly including exosomes) are bioactive vesicle-like bodies with a diameter under 200 nm that are actively secreted to the exocytosome by cells [16]. Exosomes represent a subtype of sEVs, so we use sEVs to represent exosomes herein. Because sEVs have the molecular characteristics of donor cells, they can be used as disease-specific molecular markers with substantial diagnostic value. In plasma exosomes, there are exosomes secreted by neural cells, known as plasma neurogenically derived exosomes. These exosomes are small enough to pass through the blood-brain barrier and enter the bloodstream, directly reflecting specific pathological features of the brain. They serve as an excellent carrier for reflecting brain pathology. Among plasma sEVs, there are some sEVs secreted by neural cells, known as blood neural cell-derived sEVs, which are tiny and can cross the blood-brain barrier to reach the blood. They can directly reflect the specific pathological features of the brain and serve as excellent indicators of cerebral pathology.

Several current studies assessing blood neural cell-derived sEVs for AD diagnosis and prediction hold significant clinical value. New molecules different from conventional blood diagnostic markers have been found. These blood neural cell-derived sEV molecules can not only diagnose AD, but more importantly, have important predictive value in the early stage of AD, however these studies lack systematic analysis and summary. The goal herein was to comprehensively review and summarize the changes in blood neural cell-derived sEV molecules (proteins and miRNAs), to clarify their roles in early AD prediction and diagnosis, and identify clinically valuable diagnostic biomarkers. This systematic review was registered on the International Prospective Register of Systematic Reviews (PROSPERO) with registration number CRD42024564725.

## 2 Materials and methods

This systematic review report was prepared based on the Preferred Reporting Items for Systematic Reviews and Meta-Analyses (PRISMA) 2020 statement.

### 2.1 Data sources and search strategies

Published articles were comprehensively searched via Web of Science, PubMed, Embase, and Cochrane databases through May 2024. The search terms were “AD” and “Extracellular Vesicles” and related terms, and the search formula is provided in the Supplementary Material (Appendix 1). No filter or limitation was used during the search. For all identified studies, a manual search was conducted of their references, and those in review articles, to locate additional relevant studies.

### 2.2 Inclusion and exclusion criteria

The literature inclusion criteria were:

a. The primary subjects of the study are patients with AD.
b. The sample source is blood neural cell-derived sEVs.
c. The research content focuses on blood neural cell-derived sEVs as biomarkers for the diagnosis or prediction of AD.

The exclusion criteria were:

a. Meta-analyses, reviews, and conference abstracts.
b. Literature for which the full text cannot be obtained.
c. Literature involving AD patients with other severe cerebrovascular diseases such as stroke.

### 2.3 Literature screening and information extraction

A total of 14,896 articles were retrieved. Firstly, 407 duplicate articles were excluded using EndNote X8. Subsequently, two researchers screened the articles based on their abstracts, excluding Meta-analyses, reviews, conference papers, articles whose primary research subjects were not AD, articles focusing on not blood neural cell-derived sEVs, and articles not studying AD diagnostic biomarkers, leaving 72 articles. Finally, after reviewing the full texts, articles that could not be accessed in full and those involving AD combined with other severe cerebrovascular diseases were excluded, resulting in the inclusion of 34 articles. The included articles were then carefully read again to extract relevant information, including: title, authors, country or region, year, disease type, clinical study type, basic information of included cases (diagnostic criteria, groups, age, etc.), source of neural cell-derived sEVs, methods for exosome extraction and identification, changes in sEV proteins or genes, and evaluation of clinical diagnostic value.

### 2.4 Assessment of bias risk and study quality

Agency for Health Care Research and Quality (AHRQ) [17] standards were used to evaluate cross-sectional studies (Appendix 2). The total score is 11 points, with 0–3 considered low-quality, 4–7 deemed moderate-quality, and 8–11 classified as high quality. The Newcastle-Ottawa Scale (NOS) [18] was used to assess longitudinal cohort studies (Appendix 3). With a total score of 9 points, 0–4 is considered low-quality and 5–9 indicates high-quality.

## 3 Results

### 3.1 Literature characteristics

A total of 34 articles were included herein (Table 1) [[19], [20], [21], [22], [23], [24], [25], [26], [27], [28], [29], [30], [31], [32], [33], [34], [35], [36], [37], [38], [39], [40], [41], [42], [43], [44], [45], [46], [47], [48], [49], [50], [51], [52]]. Among these, the highest rate of publication was in 2022, with seven articles (20.6%). More than half of the research came from the United States, comprising 18 articles (52.9%), followed by eight articles from China (23.5%). All 34 articles used cross-sectional study design to observe the differences between AD and other groups to find biomarkers with diagnostic value, and 11 of them included a longitudinal design to ascertain changes in related indicators and their value in predicting AD development. The AD diagnostic criteria were diverse, with the most common being the NINCDS-ADRDA (National Institute of Neurological and Communicative Disorders and Stroke and the Alzheimer's Disease and Related Disorders Association) criteria, IWG-2 criteria, and NIA-AA criteria. Eleven articles used CSF concentrations of Aβ42, t-Tau, and p-Tau181 as objective diagnostic indicators. In terms of research grouping, the primary focus was on patients with AD, including AD groups, preclinical AD (preAD) groups, MCI groups, healthy control (HC) groups, familial AD (FAD) groups, patientsfrontotemporal dementia (FTD) groups, Parkinson's disease (PD) groups, subjective cognitive decline(SCD), vascular dementia (VD) groups, and Type 2 Diabetes Mellitus (DM2) groups. Cross-sectional studies primarily compared AD and HC groups, while longitudinal studies focused on within-subjects comparisons between AD and its own preAD groups. The cumulative sample across the 34 articles was 5601 participants, including 2535 HC, 1850 AD patients, 747 MCI patients, 121 FAD patients, 108 FTD patients, 84 PD patients, 76 SCD patients, 40 VD patients and 40 DM2 patients. The average sample size for each study was 165, ranging from 29 to 739 per study. The average age range of included participants was 49–88 years, with 92.5% falling within the 60–80-year age range.

**Table 1:** Literature characteristics.

Number	Author/publication year/journal	Title	Nation	Study type	Diagnostic criteria	Groups	Sample number (M/F)	Participant age	Sample source	Neural cell-derived sEVs species	Neural cell-derived sEVs extraction method	Neural cell-derived sEVs identification method
1	Ashish Kumar et al. 2023. Alzheimers Dement	MicroRNA expression in extracellular vesicles as a novel blood-based biomarker for Alzheimer's disease	America	cross-sectional study	MoCA, GDS, CDR, ADL (USD-V3.0)	HC	11 (6/5)	71.5 ± 8.2	plasma	NDsEVs, ADsEVs, MDsEVs, ODsEVs	exosome extraction kits+immunoprecipitation (L1CAM, GLAST, TMEM119, PDGFRα)	NTA,FCM
						MCI	11 (2/9)	71.7 ± 8.3				
						MCI-AD	6 (3/3)	74.2 ± 4.4				
						AD	11 (5/6)	75.1 ± 5.9				
2	Geethu Krishna et al. 2023. J Alzheimers Dis	Pathological (Dis)Similarities in Neuronal Exosome-Derived Synaptic and Organellar Marker Levels Between Alzheimer's Disease and Frontotemporal Dementia	India	cross-sectional study	NIA-AA criteria, CDR, HMSE	HC	10 (6/4)	70	plasma	NDsEVs	exosome extraction kits+immunoprecipitation (L1CAM)	TEM,WB
						AD	9 (5/4)	58.5				
						FTD	10 (6/4)	59				
3	Chen Tian et al. 2022. Alzheimers Dement	Blood extracellular vesicles carrying synaptic function- and brain-related proteins as potential biomarkers for Alzheimer's disease	America	cross-sectional study	t-Tau/Aβ42 in CSF meets the AD standard, Aβ42=880 pg/ml, p-Tau/Aβ42=0.028, t-Tau/Aβ42=0.33	discovery cohort-AD1	45	67.59±5.59	plasma	NDsEVs	ultracentrifugation	single molecule imaging colocalization,Cryo-EM,NTA,FCM
						discovery cohort-PD	84	69.97±8.59				
						discovery cohort-HC1	32	68.74±9.48				
						validation cohort-AD2	66	75.53±8.16				
						validation cohort-HC2	82	74.04±5.65				
4	Devrim Yagmur Durur et al. 2022. J Mol Neurosci	Alteration of miRNAs in Small Neuron-Derived Extracellular Vesicles of Alzheimer's Disease Patients and the Effect of Extracellular Vesicles on Microglial Immune Responses	Turkey	cross-sectional study	DSM-IV and NINCDS-ADRDA criteria	discovery-AD	8 (4/4)	61.4 ± 6.5	plasma	NDsEVs	exosome extraction kits+immunoprecipitation (L1CAM)	NTA,WB
						discovery-HC	8 (4/4)	71.4 ± 9.4				
						validation-AD	20 (7/13)	59.3 ± 7.8				
						validation-HC	15 (10/5)	70.3 ± 7.4				
5	Erden Eren et al. 2022. Cells	Neuronal-Derived EV Biomarkers Track Cognitive Decline in Alzheimer's Disease	America	cross-sectional study	results of autopsy pathology	pure AD	21 (13/8)	76.9 ± 12.3	plasma	NDsEVs	exosome extraction kits+immunoprecipitation (L1CAM)	TEM,NTA,Exoview
						mixed AD	40 (17/23)	77.3 ± 8.5				
6	E. Taşdelen et al. 2022. Turk J Med Sci	Determination of miR-373 and miR-204 levels in neuronal exosomes in Alzheimer's disease	America	cross-sectional study	NINCDS-ADRDA criteria, CDS	mild-AD	15 (6/9)	71.40±7.76	serum	NDsEVs	exosome extraction kits+immunoprecipitation (L1CAM)	—
						moderate-AD	18 (4/14)	71.40±7.76				
						HC	21 (12/9)	66.38±6.07				
7	Tao-Ran Li et al. 2022. Alzheimers Res Ther	beta-Amyloid in blood neuronal-derived extracellular vesicles is elevated in cognitively normal adults at risk of Alzheimer's disease and predicts cerebral amyloidosis	China	cross-sectional study	MMSE, MoCA, CDR and other cognitive tests	HC (Aβ-)	84 (28/56)	65.3 ± 5.5	plasma	NDsEVs	exosome extraction kits+immunoprecipitation (L1CAM)	TEM,NTA,WB
						HC (Aβ+)	72 (24/48)	67.2 ± 6.6				
						MCI	45 (21/24)	69.6 ± 6.8				
						AD	45 (15/30)	73.9 ± 8.8				
				longitudinal cohort study		longitudinal study-HC (Aβ-)	49	The average follow-up time was 14.58±6.37 months				
						longitudinal study-HC (Aβ+)	33					
						longitudinal study-MCI	14					
						longitudinal study-AD	8					
8	X Anton Alvarez et al. 2022. J Alzheimers Dis	Modulation of Amyloid-β and Tau in Alzheimer's Disease Plasma Neuronal-Derived Extracellular Vesicles by Cerebrolysin® and Donepezil	Spain	cross-sectional study	NINCDS-ADRDA and DSM-V criteria, 12≤MMSE≤25	AD	116 (23/93)	74.87±7.55	plasma	NDsEVs	exosome extraction kits+immunoprecipitation (L1CAM)	NTA
					NINCDS-ADRDA and DSM-V criteria, MMSE>25	HC	20 (5/15)	74.35±5.76				
9	Ying Li et al. 2022. Neurobiol Dis	MicroRNA-29c-3p in dual-labeled exosome is a potential diagnostic marker of subjective cognitive decline	China	cross-sectional study	NIA-AA criteria	SCD	76 (38/38)	66.2 ± 5.8	plasma	NDsEVs	exosome extraction kits+immunoprecipitation (hamphiphysin 1 single-labeled, NCAM/amphiphysin 1 double-labeled)	TEM,NTA,WB
						MCI	80 (40/40)	65.6 ± 7.2				
						AD	80 (40/40)	66.5 ± 6.4				
						VD	40 (20/20)	65.8 ± 6.2				
						HC	40 (20/20)	65.3 ± 6.4				
10	Burak Ibrahim Arioz et al. 2021. Neurosci Lett	Proteome profiling of neuron-derived exosomes in Alzheimer's disease reveals hemoglobin as a potential biomarker	Turkey	cross-sectional study	MMSE>29	HC	23 (15/8)		serum	NDsEVs	exosome extraction kits+immunoprecipitation (L1CAM)	TEM,WB
					NINCDS-ADRDA	AD	20 (7/13)					
11	Jiacheng Zhong et al. 2021. Front Aging Neurosci	Discovery of Novel Markers for Identifying Cognitive Decline Using Neuron-Derived Exosomes	China	cross-sectional study	NINCDS-ADRDA criteria, Mini-cog, MMSE score less than 5, MMSE score ≤21 (primary school or below) or MMSE score ≤24 (secondary school or above)	discovery-AD	5		serum	NDsEVs	exosome extraction kits+immunoprecipitation (L1CAM)	TEM,NTA,ELISA
						discovery-MCI	5					
						discovery-HC	5					
						validation-AD	32					
						validation-MCI	34					
						validation-HC	52					
12	Longfei Jia et al. 2021. Alzheimers Dement	Blood neuro-exosomal synaptic proteins predict Alzheimer's disease at the asymptomatic stage	China	longitudinal cohort study	MMSE, MoCA, CDR, NIA-AA criteria	longitudinal study-AD1 (normal cognitive functionl)	160 (70/90)	60	plasma	NDsEVs	exosome extraction kits+immunoprecipitation (NCAM)	TEM,WB
						longitudinal study-AD2 (AD)	160 (70/90)	66				
						longitudinal study-HC1 (normal cognitive function)	160 (74/86)	60				
						longitudinal study-HC2 (normal cognitive function)	160 (74/86)	65				
				cross-sectional study		discovery-AD	28 (12/16)	66±6				
						discovery-MCI	25 (12/13)	65±5				
						discovery-HC	29 (14/15)	63±5				
						validation-AD	73 (31/42)	65±6				
						validation-MCI	71 (32/39)	66±7				
						validation-HC	72 (35/37)	64±5				
						familial AD (Aβ, PSEN1, and PSEN2 gene positive)	59 (29/30)	48.9				
						familial AD (Aβ, PSEN1, and PSEN2 gene negative)	62 (30/32)	48.5				
13	Pamela J. Yao et al. 2021. Biomedicines	Mitochondrial Electron Transport Chain Protein Abnormalities Detected in Plasma Extracellular Vesicles in Alzheimer's Disease	America	cross-sectional study	NIA-AA criteria, IWG-2 criteria, Aβ42<192 pg/ml and p-Tau181>23 pg/ml in CSF	AD1	22 (13/9)	73.2 ± 1.65	plasma	NDsEVs	exosome extraction kits+immunoprecipitation (L1CAM)	NTA,ELISA
						HC1	29 (17/12)	73.4 ± 2.11				
						AD2	14 (7/7)	73.1 ± 2.36				
						HC2	14 (7/7)	73.1 ± 2.11				
14	Aonan Zhao et al. 2020. Transl Neurodegener	Increased prediction value of biomarker combinations for the conversion of mild cognitive impairment to Alzheimer's dementia	China	cross-sectional study	NIA-AA criteria, MRI, MMSE, MoCA, AVLT, ADAS-cog, SAS, SDS	HC	80 (36/44)	67.3 ± 4.7	plasma	NDsEVs	exosome extraction kits+immunoprecipitation (L1CAM)	TEM,NTA,WB
						MCI	87 (40/47)	66.2 ± 4.3				
						AD	88 (38/50)	67.7 ± 4.2				
				longitudinal cohort study		longitudinal study-MCI1	70					
						longitudinal study-AD1 (2 years)	8					
						longitudinal study-MCI2	62 (28/34)	68.3 ± 4.1				
						longitudinal study-AD2 (3 years)	16 (7/9)	68.5 ± 3.8				
15	Dongmei Gu et al. 2020. Ann Clin Transl Neurol	Elevated matrix metalloproteinase-9 levels in neuronal extracellular vesicles in Alzheimer's disease	China	cross-sectional study	FDG PET, IWG-2 criteria, CDR=0.5–2.0, MMSE<27	AD	31 (8/23)	68.58± 8.04	plasma	NDsEVs	exosome extraction kits+immunoprecipitation (L1CAM)	TEM,NTA,WB,ELISA
						HC	15 (5/10)	64.80± 6.00				
16	Eunjoo Nam et al. 2020. Int J Mol Sci	Serum Tau Proteins as Potential Biomarkers for the Assessment of Alzheimer's Disease Progression	Korea	cross-sectional study	DSM-IV criteri, MMSE, CDR-SOB, GDS	HC	26 (17/9)	73.92±0.88	serum	NDsEVs	exosome extraction kits+immunoprecipitation (L1CAM)	TEM,NTA,WB
						MCI	30 (12/18)	75.13±0.99				
						AD	20 (3/17)	76.55±1.33				
17	Maria Serpente et al. 2020. Cells	MiRNA profiling in plasma neural-derived small extracellular vesicles from patients with Alzheimer’s disease	Italy	cross-sectional study	Aβ42<600 pg/ml, tau>500 pg/ml(>70 years old), 450 pg/ml (50–70 years old), p-Tau181>61 pg/ml in CSF	AD	40 (15/25)	72.8	plasma	NDsEVs	exosome extraction kits+immunoprecipitation (L1CAM)	TEM,NTA,WB
						HC	40 (22/18)	66.6				
18	Nan Zhang et al. 2020. Front Aging Neurosci	TDP-43 Is Elevated in Plasma Neuronal-Derived Exosomes of Patients With Alzheimer’s Disease	China	cross-sectional study	IWG-2 criteria, PET, CDR, MMSE, NPI	HC	15 (5/10)	64.8 ± 6.0	plasma	NDsEVs	exosome extraction kits+immunoprecipitation (L1CAM)	TEM,NTA
						AD	24 (7/17)	67.8 ± 8.2				
19	Charisse N. Winston et al. 2019. Alzheimers Dement (Amst)	Complement protein levels in plasma astrocyte-derived exosomes are abnormal in conversion from mild cognitive impairment to Alzheimer's disease dementia	America	cross-sectional study	Aβ42<192pg/ml in CSF, IWG-2 criteria, CDR=0.5–1.0, MMSE	HC	20 (12/8)	70.8 ± 5.34	plasma	ADsEVs	exosome extraction kits+immunoprecipitation (GLAST)	—
						MCI	20 (13/7)	68.7 ± 7.76				
						MCI-AD	20 (11/9)	75.4 ± 6.82				
						AD	20 (12/8)	71.1 ± 6.90				
20	Cristina Agliardi et al. 2019. Mol Neurobiol	SNAP-25 in Serum Is Carried by Exosomes of Neuronal Origin and Is a Potential Biomarker of Alzheimer's Disease	Italy	cross-sectional study	NINCDS-ADRDA criteria, DSM-IV-TR, MMSE	HC	17 (4/13)	76.47±6.16	serum	NDsEVs	exosome extraction kits+immunoprecipitation (L1CAM)	WB
						AD	24 (8/16)	77.67±6.84				
21	Diana J Cha et al. 2019. Front Neurosci	miR-212 and miR-132 Are Downregulated in Neurally Derived Plasma Exosomes of Alzheimer's Patients	America	cross-sectional study	p-Tau181 and Aβ in CSF, Aβ, MMSE	HC	16		plasma	NDsEVs	exosome extraction kits+immunoprecipitation (L1CAM)	—
						MCI	16					
						AD	31					
22	Dimitrios Kapogiannis et al. 2019. JAMA Neurol	Association of Extracellular Vesicle Biomarkers With Alzheimer Disease in the Baltimore Longitudinal Study of Aging	America	longitudinal cohort study	BIMC test, CDR, NINCDS-ADRDA criteria	longitudinal study-HC (baseline)	222 (112/110)	76.02	plasma, serum	NDsEVs	exosome extraction kits+immunoprecipitation (L1CAM)	TEM,NTA
						longitudinal study-HC (norma cognitive functionl)	94					
						longitudinal study-AD	128 (60/68)	79.09				
				cross-sectional study		HC	35 (17/18)	74.03				
						AD	29 (6/23)	72.14				
23	Longfei Jia et al. 2019. Alzheimers Dement	Concordance between the assessment of Aβ42, T-tau, and P-T181-tau in peripheral blood neuronal-derived exosomes and cerebrospinal fluid	China	cross-sectional study	NIA-AA criteria	discovery-AD	28 (12/16)	66±6	plasma	NDsEVs	exosome extraction kits+immunoprecipitation (NCAM)	TEM,WB
						discovery-MCI	25 (12/13)	65±5				
						discovery-HC	29 (14/15)	63±5				
						validation-AD	73 (31/42)	65±6				
						validation-MCI	71 (32/39)	66±7				
						validation-HC	72 (35/37)	64±5				
24	Charisse N Winston et al. 2018. J Alzheimers Dis	Growth Hormone-Releasing Hormone Modulation of Neuronal Exosome Biomarkers in Mild Cognitive Impairment	America	cross-sectional study	NIA criteria	HC	76 (29/47)	67.8 ± 2.3	plasma	NDsEVs	exosome extraction kits+immunoprecipitation (L1CAM)	—
						MCI	61 (22/39)	70.2 ± 2.3				
25	Edward J Goetzl et al. 2018. FASEB J	Declining levels of functionally specialized synaptic proteins in plasma neuronal exosomes with progression of Alzheimer's disease	America	cross-sectional study	Aβ42<192 pg/ml and p-Tau181>23 pg/ml in CSF, NIA criteria, IWG-2 criteria	HC	28 (12/16)	73.2 ± 1.47	plasma	NDsEVs	exosome extraction kits+immunoprecipitation (L1CAM)	—
						AD	28 (12/16)	73.1 ± 1.44				
				longitudinal cohort study		longitudinal study-HC	18 (10/8)	70.1 ± 0.66				
						longitudinal study-AD1 (normal cognitive function)	18 (10/8)	69.4 ± 1.71				
						longitudinal study-AD2 (AD)	18 (10/8)	78.2 ± 1.75				
26	Edward J. Goetzl et al. 2017. Ann Neurol	High complement levels in astrocyte-derived exosomes of Alzheimer disease	America	cross-sectional study	Aβ42<192pg/ml, p-Tau181>23pg/ml in CSF, NIA-AA criteria, IWG-2 criteria, MMSE, ADAS-cog, CDR=0.5–1.0	cross-sectional study-HC	28 (12/16)	73.2 ± 1.47	plasma	ADsEVs	exosome extraction kits+immunoprecipitation (GLAST)	—
						cross-sectional study-AD	28 (12/16)	73.1 ± 1.44				
				longitudinal cohort study		longitudinal study-HC	16 (9/7)	69.7 ± 2.04				
						longitudinal study-AD1 (normal cognitive function)	16 (9/7)	70.3 ± 1.75				
						longitudinal study-AD2 (5–12y after AD)	16 (9/7)	79.1 ± 1.83				
27	Charisse N Winston et al. 2016. Alzheimers Dement (Amst)	Prediction of conversion from mild cognitive impairment to dementia with neuronally derived blood exosome protein profile	America	cross-sectional study	CSF Aβ42 <190 pg/ml,MMSE <20	HC	10		plasma	NDsEVs	exosome extraction kits+immunoprecipitation (L1CAM)	TEM,NTA
						MCI	20(13/7)	68.7 ± 7.76				
						MCI-AD	20(11/9)	75.35±6.82				
						AD	10					
28	Edward J Goetzl et al. 2016. FASEB J	Cargo proteins of plasma astrocyte-derived exosomes in Alzheimer's disease	America	cross-sectional study	Aβ42<192pg/ml in CSF, CDR, MMSE, ADAS-cog	AD	12		plasma	ADsEVs, NDsEVs	exosome extraction kits+immunoprecipitation (GLAST)	NTA
						HC	10					
						FTD	14					
						FTC	10					
29	Edward J Goetzl et al. 2016. FASEB J	Decreased synaptic proteins in neuronal exosomes of frontotemporal dementia and Alzheimer's disease	America	cross-sectional study	NIA criteria, NINCDS-ADRDA criteria, CSF Aβ42, CDR, MMSE, ADAS-cog	AD	12 (6/6)	74.4 ± 1.98	plasma	NDsEVs	exosome extraction kits+immunoprecipitation (L1CAM)	—
						HC	12 (6/6)	74.4 ± 1.98				
						FTD	16 (12/4)	63.6 ± 1.82				
						FTC	16 (12/4)	63.6 ± 1.82				
				longitudinal cohort study		longitudinal study-HC	9 (2/7)	81.7 ± 2.06				
						longitudinal study-AD1 (normal cognitive function)	9 (2/7)	82.2 ± 2.28				
						longitudinal study-AD2 (AD)	9 (2/7)	87.8 ± 2.50				
						longitudinal study-FTC	10 (5/5)	63.6 ± 2.04				
						longitudinal study-FTD1 (normal cognitive function)	10 (5/5)	62.9 ± 1.93				
						longitudinal study-FTD2 (FTD)	10 (5/5)	66.5 ± 1.82				
30	Erin L. Abner et al. 2016. Ann Clin Transl Neurol	Plasma neuronal exosomal levels of Alzheimer's disease biomarkers in normal aging	England	longitudinal cohort study	ADAS-cog, neurological examination, psychological test, CSF protein, MRI	longitudinal study-time1 (normal cognitive function)	18 (8/10)	69.3 ± 5.6	plasma	NDsEVs	exosome extraction kits+immunoprecipitation (L1CAM)	—
						longitudinal study-time2 (3–11years later)	20 (10/10)	77.6 ± 6.1				
				cross-sectional study	Aβ42<192pg/ml in CSF	HC	10					
						AD	20 (10/10)	77.6 ± 6.1				
31	Dimitrios Kapogiannis et al. 2015. FASEB J	Dysfunctionally phosphorylated type 1 insulin receptor substrate in neural-derived blood exosomes of preclinical Alzheimer's disease	America	cross-sectional study	Aβ42<192 pg/ml in CSF, CDR, MMSE, ADAS-cog	AD	26 (13/13)	74.3 ± 7.48	plasma	NDsEVs	exosome extraction kits+immunoprecipitation (L1CAM)	—
						HC	26 (13/13)	74.3 ± 7.48				
						FTD	16 (12/4)	63.1 ± 8.79				
						FTC	16 (12/4)	63.7 ± 7.43				
						DM2	20 (9/11)	73.0 ± 9.27				
						DC	20 (9/11)	73.0 ± 9.19				
				longitudinal cohort study		longitudinal study-AD1 (normal cognitive function)	22 (12/10)					
						longitudinal study-AD2 (AD)	22 (12/10)	78.0 ± 1.69				
32	Edward J Goetzl et al. 2015. Neurology	Altered lysosomal proteins in neural-derived plasma exosomes in preclinical Alzheimer disease	America	cross-sectional study	Aβ42<192 pg/ml in CSF, NIA criteria, NINCDS-ADRDA criteria, CDR, MMSE	AD	26 (13/13)	75.4 ± 7.85	plasma	NDsEVs	exosome extraction kits+immunoprecipitation (L1CAM)	—
						HC	26 (13/13)	75.8 ± 7.91				
						FTD	16 (12/4)	63.1 ± 8.79				
						FTC	16 (12/4)	63.7 ± 7.43				
				longitudinal cohort study		longitudinal study-HC	20 (10/10)	73.8 ± 5.93				
						longitudinal study-AD1 (normal cognitive functionl)	20 (10/10)	74.0 ± 5.95				
						longitudinal study-AD2 (AD)	20 (10/10)	80.1 ± 6.21				
33	Edward J. Goetzl et al. 2015. Ann Clin Transl Neurol	Low neural exosomal levels of cellular survival factors in Alzheimer's disease	America	cross-sectional study	ADAS-cog, NIA-AA criteria, IWG-2 criteria	AD	24 (12/12)	75.7 ± 7.59	plasma	NDsEVs	exosome extraction kits+immunoprecipitation (L1CAM)	—
						HC	24 (12/12)	75.1 ± 7.18				
						FTD	10 (7/3)	64.5 ± 9.13				
						FTC	10 (7/3)	64.2 ± 8.76				
						DM2	20 (9/11)	73.0 ± 9.27				
						DC	20 (9/11)	73.0 ± 9.19				
				longitudinal cohort study		longitudinal study-HC	16 (7/9)	78.3 ± 5.99				
						longitudinal study-AD1 (normal cognitive function)	16 (7/9)	78.2 ± 6.44				
						longitudinal study-AD2 (AD)	16 (7/9)	83.8 ± 7.54				
34	Massimo S. Fiandaca et al. 2015. Alzheimers Dement	Identification of preclinical Alzheimer's disease by a profile of pathogenic proteins in neurally derived blood exosomes: A cross-sectional study	America	cross-sectional study	NINCDS-ADRDA criteria, MMSE	AD	57 (30/27)	79.5 ± 6.05	plasma, serum	NDsEVs	exosome extraction kits+immunoprecipitation (L1CAM)	—
						HC	57 (30/27)	79.6 ± 6.03				
						FTD	16 (12/4)	63.1 ± 8.79				
						FTC	16 (12/4)	63.7 ± 7.43				
				longitudinal cohort study		longitudinal study-AD1 (normal cognitive functionl)	24 (12/12)	71.8 ± 7.30				
						longitudinal study-AD2 (AD)	24 (12/12)					

AD: Alzheimer's disease; ADAS-cog: Alzheimer's disease assessment scale-cognitive subscale; ADL: activity of daily living scale; ADsEVs: astrocyte-derived small extracellular vesicles; BIMC: blessed information memory concentration; CDR: clinical dementia rating; CDS: carroll depression scales; Cryo-EM: cryoelectron microscopy; CSF: cerebrospinal fluid; DM2: type 2 diabetes mellitus; DSM: the diagnostic and statistical manual of mental disorders; DSM-IV-TR:diagnostic and statistical manual of mental disorders, fourth edition, text revision; ELISA: enzyme-Linked immunosorbent assay; FCM: flow cytometry;FDG: 18F-fluorodeoxyglucose; FTC: frontotemporal dementia control; FTD: frontotemporal dementia; GDS: geriatric depression scale; GLAST: glutamate-aspartate transporter; HC: healthy control; HMSE: hindi mental state examination; IWG: international working group; L1CAM: L1 cell adhesion molecule; MCI: mild cognitive impairment; MDsEVs: microglia-derived small extracellular vesicles; MMSE: mini mental state examination scale; MoCA: montreal cognitive assessmen; MRI: magnetic resonance imaging; NDsEVs: neuron-derived small extracellular vesicles; NIA-AA: National Institute on Aging-Alzheimer's Association; NINCDS-ADRDA: national institute of neurological and communicative disorders and stroke and the Alzheimer's disease and related disorders association; NPI: neuropsychiatric inventory; NTA: nanoparticle tracking analysis; ODsEVs: oligodendrocyte-derived small extracellular vesicles; PD: Parkinson's disease; PDGFRα: platelet-derived growth factor receptor alpha; PET: positron emission tomography; PSEN: presenilin; SCD: subjective cognitive decline; sEVs: small extracellular vesicles; TEM: transmission electron microscope; TMEM119: transmembrane protein 119; VD: vascular dementia; WB: western blotting.

All 34 studies involved sEVs derived from blood, primarily from plasma (in 27 articles, 79.4%) and from serum in five articles (14.7%); two articles included plasma and serum, but without specifying which groups used plasma versus serum. In terms of neural cell-derived sEV types, 31 articles (91.2%) focused on neuron-derived sEVs (NDsEVs), three (8.8%) on astrocyte-derived sEVs (ADsEVs), and one on sEVs from multiple sources, including NDsEVs and glial cell-derived sEVs. Glial cell-derived sEVs includes ADsEVs, microglia-derived sEVs (MDsEVs), and oligodendrocyte-derived sEVs (ODsEVs). Regarding the method of extracting sEVs, 32 studies used exosome extraction kits, then enriched neural cell-derived sEVs by immunoprecipitation with antibodies to characterize the proteins of neurons or glial cells. Only one study used ultracentrifugation for extracting neural cell-derived sEVs. The sEV identification mainly relied on transmission electron microscope (TEM), nanoparticle tracking analysis (NTA), western blot (WB), and flow cytometry.

### 3.2 Literature quality evaluation

Among the 34 cross-sectional studies, 20 (58.8%) were high-quality, 13 (41.2%) were moderate-quality, and none were low-quality. Their average score was 7.0 points (out of a total 11 points possible). All 11 longitudinal cohort studies were high-quality, with an average score of 6.45 points (out of a total of 9 points possible).

### 3.3 Cross-sectional study results

Cross-sectional study results are presented in Tables 2, 3, and 4. 2 contains the NDsEVs results, representing 31 articles. Table 3 presents glial cell-derived sEV results from four articles, including ADsEVs, MDsEVs, and ODsEVs. Table 4 categorizes the neural cell-derived sEV molecules (proteins and miRNA) from these cross-sectional studies.

**Table 2:** Blood NDsEV changes in patients with AD, compared with HC, from cross-sectional studies.

Number	Author/publication year	Groups (sample size)	Literature quality	Changes in NDsEVs, compared with HC	Results
1	Ashish Kumar et al. 2023	HC (11) MCI (11) MCI-AD (6) AD (11)	high-quality	miR–9–5p↑, miR-106b-5p↑, miR-125b-5p↑, miR–132–5p↑, miR-29a-5p↓	Compared with HC, plasma NDsEV miR–9–5p, miR-106b-5p, miR-125b-5p, and miR–132–5p increased significantly, but miR-29a-5p decreased significantly in patients with AD.
2	Geethu Krishna et al. 2023	HC (10) AD (9) FTD (10)	moderate-quality	neurogranin↓, MFN-2↓, LAMP-2↑, golgin A4↑	Compared with HC, plasma NDsEV LAMP-2 and golgin A4 increased significantly, but neurogranin and MFN-2 decreased significantly in patients with AD.
3	Chen Tian et al. 2022	discovery cohort-AD1 (45) discovery cohort-PD (84) discovery cohort-HC1 (32) validation cohort-AD2 (66) validation cohort-HC2 (82)	high-quality	NMDAR2A↓, L1CAM↓, Aβ40, Aβ42↓, p-Tau231, p-Tau396↓	Compared with HC, plasma NDsEV NMDAR2A, L1CAM, Aβ40, Aβ42, p-Tau231, and p-Tau396 decreased significantly in patients with AD.
4	Devrim Yagmur Durur et al. 2022	discovery-AD (8) discovery-HC (8) validation-AD (20) validation-HC (15)	moderate-quality	let-7e-5p↑, miR–96–5p↑, miR-484↑, miR-99b-5p↓, miR–100–5p↓, miR-30e-5p↓, miR-378i↓, miR–145–5p↓, miR-378c↓, miR-451a↓	Compared with HC, plasma NDsEV let-7e-5p, miR–96–5p, and miR-484 increased significantly, but miR-99b-5p, miR–100–5p, miR-30e-5p, miR-378i, miR–145–5p, miR-378c, and miR-451a decreased significantly in patients with AD.
5	Erden Eren et al. 2022	pure AD (21) mixed AD (40)	moderate-quality	Aβ42 (no change), p-Tau181 (no change), t-Tau (no change), synaptophysin (no change), synaptopodin(no change)	There was no difference between two groups.
6	E. Taşdelen et al. 2022	mild-AD (15) moderate-AD (18) HC (21)	high-quality	miR-204↓, miR-373↓	Compared with HC, serum NDsEV miR-204 and miR-373 decreased significantly in patients with AD.
7	Tao-Ran Li et al. 2022	HC (Aβ-) (84) HC (Aβ+) (72) MCI (45) AD (45)	high-quality	Aβ40↑, Aβ42↑	Compared with HC(Aβ+) and HC(Aβ-), plasma NDsEV Aβ42 and Aβ40 significantly in patients with AD.
8	X Anton Alvarez et al. 2022	HC (20) AD (116)	high-quality	Aβ42↑, t-Tau↑, p-Tau181↑ (AD) p-S396-Tau↑ (mild to moderate AD) neurogranin↓, REST↓ (mild to moderate AD)	Compared with HC, plasma NDsEV Aβ42, t-Tau, p-Tau181 and p-S396-Tau increased significantly, but neurogranin and REST decreased significantly in patients with mild to moderate AD.
9	Ying Li et al. 2022	SCD (76) MCI (80) AD (80) VD (40) HC (40)	high-quality	Aβ42↑, Aβ42/40↑, t-Tau↑, p-Tau181↑, NfL↑, miR-29c-3p↑	① Amphiphysin 1 single-labeled:Compared with HC, plasma NDsEV Aβ42/40, t-Tau, p-Tau181, NfL, and miR-29c-3p increased significantly in patients with ADI. ② NCAM/amphiphysin 1 double-labeled:Compared with HC, plasma NDsEV Aβ42, Aβ42/40, t-Tau, p-Tau181, and NfL increased significantly in patients with AD.
10	Burak Ibrahim Arioz et al. 2021	HC (23) AD (20)	high-quality	Alpha-globin↑, Beta-globin↑, Delta-globin↑	Compared with HC, serum NDsEV Alpha-globin, Beta-globin, and Delta-globin increased significantly in patients with AD.
11	Jiacheng Zhong et al. 2021	discovery-AD (5) discovery-MCI (5) discovery-HC (5) validation-AD (32) validation-MCI (34) validation-HC (52)	moderate-quality	C7↑, ZYX↓	Compared with HC, serum NDsEV C7 increased significantly, but ZYX decreased in patients with AD.
12	Longfei Jia et al. 2021	discovery-AD (28) discovery-MCI (25) discovery-HC (29) validation-AD (73) validation-MCI (71) validation-HC (72)	high-quality	GAP43↓, neurogranin↓, SNAP25↓, synaptotagmin 1↓	Compared with HC, plasma NDsEV GAP43, neurogranin, SNAP25, and synaptotagmin 1 decreased significantly in patients with AD.
13	Pamela J. Yao et al. 2021	AD1 (22) HC1 (29) AD2 (14) HC2 (14)	moderate-quality	ATP synthase↓, SOD1↓, complex I (1)↓, complex I (6)↓, complex III↓, complex IV↓	Compared with HC, plasma NDsEV ETC complexes, ATPsynthase, SOD1, complex I (1), complex I (6), complex III and complex IV decreased significantly in patients with AD.
14	Aonan Zhao et al. 2020	HC (80) MCI (87) AD (88)	high-quality	Aβ42↑, Aβ42/40↑, SS-16 scores↓	Compared with HC, plasma NDsEV Aβ42 and Aβ42/40 increased significantly in patients with AD, and the SS-16 scores of patients with AD decreased significantly.
15	Dongmei Gu et al. 2020	AD (31) HC (15)	high-quality	MMP-9↑, Aβ42↑, p-Tau181↑, p-S396-Tau (no change), IL-6 (no change)	Compared with HC, plasma NDsEV Aβ42, p-Tau181 and MMP-9 increased significantly in patients with AD.
16	Eunjoo Nam et al. 2020	HC (26) MCI (30) AD (20)	moderate-quality	p-Tau↑, t-Tau↑	Compared with HC, serum NDsEV p-Tau and t-Tau increased significantly in patients with AD.
17	Maria Serpente et al. 2020	AD (40) HC (40)	high-quality	miR-23a-3p↑, miR–223–3p↑, miR-190a-5p↑, miR–100–3p↓,	Compared with HC, plasma NDsEV miR-23a-3p, miR–223–3p and miR-190a-5p increased significantly, but miR–100–3p decreased in patients with AD.
18	Nan Zhang et al. 2020	HC (15) AD (24)	high-quality	TDP-43↑	Compared with HC, plasma NDsEV TDP-43 increased significantly in patients with AD.
19	Cristina Agliardi et al. 2019	HC (17) AD (24)	moderate-quality	SNAP25↓	Compared with HC, serum NDsEV SNAP25 decreased significantly in patients with AD.
20	Diana J Cha et al. 2019	HC (16) MCI (16) AD (31)	moderate-quality	miR–132–3p↓, miR–212–3p↓	Compared with HC, plasma NDsEV miR–132–3p and miR–212–3p decreased significantly in patients with AD.
21	Dimitrios Kapogiannis et al. 2019	HC (35) AD (29)	high-quality	p-Tau231↑, p-Tau181↑, p-S312-IRS-1↑, pY-IRS-1↑	Compared with HC, plasma NDsEV p-Tau181, p-Tau231, p-S312-IRS-1 and pY-IRS-1 increased significantly in patients with AD.
22	Longfei Jia et al. 2019	discovery-AD (28) discovery-MCI (25) discovery-HC (29) validation-AD (73) validation-MCI (71) validation-HC (72)	moderate-quality	Aβ42↑, t-Tau↑, p-Tau181↑	Compared with HC, plasma NDsEV Aβ42, t-Tau, and p-Tau181 increased significantly in patients with AD.
23	Charisse N Winston et al. 2018	HC (76) MCI (61)	moderate-quality	Aβ42↑, neurogranin↓, synaptophysin↓, synaptotagmin↓, synaptopodin↓	Compared with HC, plasma NDsEV Aβ42 increased, but neurogranin, synaptophysin, synaptotagmin and synaptopodin decreased significantly in patients with MCI.
24	Edward J Goetzl et al. 2018	HC (28) AD (28)	high-quality	AMPA4↓, NPTX2↓, NLGN1↓, NRXN2α↓	Compared with HC, plasma NDsEV AMPA4, NPTX2, NLGN1 and NRXN2α decreased significantly in patients with AD.
25	Charisse N Winston et al. 2016	HC (10) MCI (20) MCI-AD (20) AD (10)	moderate-quality	Aβ42 ↑, p-Tau181 ↑, p-S396-Tau ↑, neurogranin ↓, REST ↓	Compared with HC, plasma NDsEV Aβ42, p-Tau181 and p-S396-Tau increased, but neurogranin and REST decreased significantly in patients with AD.
26	Edward J Goetzl et al. 2016	AD (12) HC (12) FTD (16) FTC (16)	high-quality	synaptotagmin↓, synaptopodin↓, synaptophysin↓, neurogranin↓, GAP43↓	Compared with HC, plasma NDsEV synaptotagmin, synaptopodin, synaptophysin, neurogranin, and GAP43 decreased significantly in patients with AD.
27	Erin L. Abner et al. 2016	AD (10) HC (10)	moderate-quality	Aβ42, p-Tau181, p-S396-Tau↑Cathepsin D↑, REST↓, neurogranin↓	Compared with HC, plasma NDsEV Aβ42, p-Tau181, p-S396-Tau and Cathepsin D increased, but REST and neurogranin decreased significantly in patients with AD.
28	Dimitrios Kapogiannis et al. 2015	AD (26) HC (26) FTD (16) FTC (16) DM2 (20) DC (20)	high-quality	p-panY-IRS-1↓, p-S312-IRS-1↑, p-S312-IRS-1/p-panY-IRS-1↑	Compared with HC, plasma NDsEV p-S312-IRS-1 and p-S312-IRS-1/p-panY-IRS-1 increased, but p-panY-IRS-1 decreased significantly in patients with AD.
29	Edward J Goetzl et al. 2015	AD (26) HC (26) FTD (16) FTC (16)	high-quality	cathepsin D↑, LAMP-1↑, ubiquitin↑, HSP70↓	Compared with HC, plasma NDsEV cathepsin D, LAMP-1 and ubiquitin increased, but HSP70 decreased significantly in patients with AD.
30	Edward J. Goetzl et al. 2015	AD (24) HC (24) FTD (10) FTC (10)	high-quality	LRP6↓, HSF1↓, REST↓	Compared with HC, plasma NDsEV LRP6, HSF1 and REST decreased significantly in patients with AD.
31	Massimo S. Fiandaca et al. 2015	AD (57) HC (57) FTD (16) FTC (16)	high-quality	t-Tau↑, p-S396-Tau↑, p-Tau181↑, Aβ42↑	Compared with HC, blood NDsEV Aβ42, t-Tau, p-Tau181 and p-S396-Tau increased significantly in patients with AD.

AD: Alzheimer's disease; AMPA4: glua4-containing glutamate; ATP: adenosine triphosphate; CSF: cerebrospinal fluid; DM2: type 2 diabetes mellitus; ETC: electron transport chain; FTC: frontotemporal dementia control; FTD: frontotemporal dementia; HC: healthy control; HSF1: heat shock factor 1; HSP70: heat-shock protein 70; IL-6: interleukin-6; L1CAM: L1 cell adhesion molecule; LAMP: lysosome-associated membrane protein; LRP6: lipoprotein receptor-related protein 6; MCI: mild cognitive impairment; MFN-2: mitofusin-2; MMP-9: matrix metalloproteinase-9; NDsEVs: neuron-derived small extracellular vesicles; NfL: neurofilament light chain; NLGN1: neuroligin1; NMDAR2A: N-methyl-d-aspartate receptor 2A; NPTX2: neuronal pentraxin 2; NRXN2α: neurexin 2α; PD: Parkinson's disease; REST: repressor element 1-silencing transcription factor; SCD: subjective cognitive decline; SNAP25: ‌synaptosomal-associated protein, 25 kDa; SOD1: ‌superoxide dismutase 1; SS-16: sniffin sticks 16; TDP-43: TAR DNA binding protein of 43 kDa; VD: vascular dementia.

**Table 3:** Blood glial cell-derived sEV changes in patients with AD, compared with HC, from cross-sectional studies.

Number	Author/publication year	Groups (sample size)	Literature quality	Changes in glial cell-derived sEVs, compared with HC	Results
1	Ashish Kumar et al. 2023	HC (11) MCI (11) MCI-AD (6) AD (11)	high-quality	ADsEVs:miR-29a-5p↑, miR-125b-5p↑, miR–132–5p↑, miR-107↑ MDsEVs:miR-29a-5p↓, miR-125b-5p↑, miR–132–5p↑, miR-106b-5p↑, miR-107↑, miR–210–3p↑ ODsEVs:miR-29a-5p↑, miR-107↑, miR-135b-5p↑, miR-125b-5p↑	ADsEVs:Compared with HC, plasma ADsEV miR-29a-5p, miR-125b-5p, miR–132–5p and miR-107 increased significantly in patients with AD. MDsEVs:Compared with HC, plasma MDsEV miR-125b-5p, miR–132–5p, miR-107, miR-106b-5p and miR–210–3p increased, but miR-29a-5p decreased significantly in patients with AD. ODsEVs:Compared with HC, plasma ODsEV miR-29a-5p, miR-107, miR-125b-5p and miR-135b-5p increased significantly in patients with AD.
2	Charisse N. Winston et al. 2019	HC (20) MCI (20) MCI-AD (20) AD (20)	moderate-quality	C1q↑, C4b↑, Factor D↑, Bb↑, C5b↑, C3b↑, C5b-C9 TCC↑, DAF↓, CD46↓, CD59↓, CR1↓	Compared with MCI, plasma ADsEV C1q, C4b, Factor D, fragment Bb, C5b, C3b and C5b-C9 increased, but DAF, CD46, CD59 and CR1 decreased significantly in patients with AD.
3	Edward J. Goetzl et al. 2017	HC (28) AD (28)	high-quality	C1q↑, C4b↑, C3d↑, Factor B↑, Factor D↑, Bb↑, C3b↑, C5b-C9 TCC↑, IL-6↑, TNF-α↑, IL-1β↑, CD59↓, DAF↓, CD46↓, CR1↓	Compared with HC, plasma ADsEV complement effect proteins C1q, C4b, C3d, Factor B, Factor D, Bb, C3b, and TCC increased, inflammatory cytokines IL-6, TNF-α, and IL-1βincreased, but complement regulatory proteins CD59, DAF, CD46, and CR1 decreased significantly in patients with AD. Compared with HC, plasma NDsEV TNF-α decreased significantly in patients with AD.
4	Edward J Goetzl et al. 2016	AD (12) HC (10) FTD (14) FTC (10)	moderate-quality	BACE-1↑, sAPPβ↑, GDNF↓	Compared with HC, plasma ADsEV BACE-1 and sAPPβ increased, but GDNF decreased significantly in patients with AD.

AD: Alzheimer's disease; ADsEVs: astrocyte-derived small extracellular vesicles;BACE-1: β-site amyloid precursor protein-cleaving enzyme 1; CD46: cluster of differentiation 46 protein; CD59: cluster of differentiation 59 protein; CR1: complement receptor type 1; DAF: decay-accelerating factor;FTC: frontotemporal dementia control; FTD: frontotemporal dementia; GDNF: glial-derived neurotrophic factor;HC: healthy control; MCI: mild cognitive impairment; MDsEVs: microglia-derived small extracellular vesicles; NDsEVs: neuron-derived small extracellular vesicles; ODsEVs: oligodendrocyte-derived small extracellular vesicles; sAPP: soluble amyloid precursor protein; TCC: terminal complement complex.

**Table 4:** Classification of and changes in blood neural cell-derived sEVs in patients with AD, compared with HC, from cross-sectional studies.

Classification of sEV molecules	Changes of sEV molecules, compared with HC	sEV molecules
Aß-related proteins	Increase↑	Aβ42(*10), Aβ42/40(*2), Aβ40, BACE-1△, sAPPβ△
	Decrease↓	Aβ42, Aβ40
	No change	
Tau-related proteins	Increase↑	p-Tau181(*8), t-Tau(*5), p-S396-Tau(*3), p-Tau, p-Tau231
	Decrease↓	p-Tau231, p-Tau396
	No change	p-S396-Tau
Synaptic related proteins	Decrease↓	neurogranin(*6), synaptotagmin(*2), synaptophysin(*2), synaptopodin(*2), GAP43(*2), SNAP25(*2), synaptotagmin 1, NMDAR2A, L1CAM, AMPA4, NPTX2, NLGN1, NRXN2α
Immunoinflammatory-related proteins	Increase↑	MMP-9, Alpha-globin, Beta-globin, Delta-globin, C7, C1q(*2)△, C4b(*3)▲, Factor D(*3)▲, Bb(*3)▲, C3b(*2)△, C5b△, C5b-C9 TCC(*3)▲, C3d(*2)▲, Factor B(*2)▲, IL-6△, TNF-α△, IL-1β△
	Decrease↓	ZYX, HSP70, TNF-α, DAF(*2)△, CD46(*2)△, CD59(*2)△, CR1(*2)△
	No change	IL-6
miRNAs	Increase↑	miR-125b-5p(*4)▲⬛★, miR–132–5p(*3)▲⬛, miR-106b-5p⬛, miR-107(*3)△□☆, miR-29a-5p(*2)△☆, miR–210–3p□, miR-135b-5p☆, miR–9–5p, miR-23a-3p, miR-29c-3p, miR–96–5p, miR-190a-5p, miR–223–3p, miR-484, let-7e-5p
	Decrease↓	miR-29a-5p(*2)⬛, miR-30e-5p, miR-99b-5p, miR–100–3p, miR–100–5p, miR–132–3p, miR–145–5p, miR-204, miR–212–3p, miR-373, miR-378i, miR-378c, miR-451a
Others	Increase↑	Brain insulin resistance related proteins:p-S312-IRS-1(*2), pY-IRS-1, p-S312-IRS-1/p-panY-IRS-1 Lysosome-related proteins:LAMP-1, LAMP-2, Cathepsin D, ubiquitin
	Decrease↓	Energy metabolism related proteins: ATP synthase, complex I(1), complex I(6), complex III, complex IV, MFN-2 Lysosome-related proteins: p-panY-IRS-1 Oxidative stress-related protein: SOD1 Transcription-related proteins: REST(*3), LRP6, HSF1, TDP-43 Neurotrophic factor: GDNF△

△: Just detect the ADsEV molecules; ▲: Detect both ADsEV and NDsEV molecules; □: Just detect the MDsEV molecules; ⬛: Detect both MDsEV and NDsEV molecules; ☆: Just detect the ODsEV molecules; ★: Detect both ODsEV and NDsEV molecules.

p-Tau181(*7): There were seven studies about p-Tau181 herein.

AD: Alzheimer's disease; ADsEVs: astrocyte-derived small extracellular vesicles; AMPA4: glua4-containing glutamate; ATP: adenosine triphosphate; BACE-1: β-site amyloid precursor protein-cleaving enzyme 1; CD46: cluster of differentiation 46 protein; CD59: cluster of differentiation 59 protein; CR1: complement receptor type 1; DAF: decay-accelerating factor; GAP43: growth associated protein 43; GDNF: glial-derived neurotrophic factor;HSF1: heat shock factor 1; HSP70: heat-shock protein 70; IL-1β: interleukin-1β; IL-6: interleukin-6; L1CAM: L1 cell adhesion molecule; LAMP: lysosome-associated membrane protein; LRP6: lipoprotein receptor-related protein 6; MDsEVs: microglia-derived small extracellular vesicles; MFN-2: mitofusin-2; MMP-9: matrix metalloproteinase-9; NDsEVs: neuron-derived small extracellular vesicles; NLGN1: neuroligin1; NMDAR2A: N-methyl-d-aspartate receptor 2A; NPTX2: neuronal pentraxin 2; NRXN2α: neurexin 2α; ODsEVs: oligodendrocyte-derived small extracellular vesicles; REST: repressor element 1-silencing transcription factor; sAPP: soluble amyloid precursor protein; SNAP25: ‌synaptosomal-associated protein, 25 kDa; SOD1: ‌superoxide dismutase 1; TCC: terminal complement complex; TDP-43: TAR DNA binding protein of 43 kDa; TNF-α: tumor necrosis factor-α; ZYX: zyxin.

Table 2 summarizes differences in neural cell-derived sEVs, between patients with AD and matched controls to assess the diagnostic value of neural cell-derived sEVs. Combined with the results in Table 4, neural cell-derived sEVs with significant differences between AD and HC groups included Aβ-related proteins, Tau-related proteins, synaptic-related proteins, immunoinflammatory-related proteins, miRNA molecules, and other protein categories. Among these, most Aβ-related proteins exhibited changes consistent with the classical theory, with significant increases in Aβ42, Aβ42/40, and Aβ40; however, one study described decreases in Aβ42 and Aβ40 in AD [21]. Tau-related proteins p-Tau181, t-Tau, p-S396-Tau, p-Tau, and p-Tau231 mostly increased in AD, though one study indicated decreases in p-Tau231 and p-Tau396 in patients with AD [21] and another study showed no significant changes in p-S396-Tau in AD [33]. Synaptic-related proteins neurogranin, synaptotagmin, synaptotagmin-1, synaptophysin, synaptopodin, GAP43, SNAP25, NMDAR2A, L1CAM, AMPA4, NPTX2, NLGN1, and NRXN2α exhibited significant decreases in patients with AD. Immunoinflammatory-related proteins MMP-9, Alpha-globin, Beta-globin, Delta-globin, and C7 showed significant increases, while complement effect proteins C4b, Factor D, fragment Bb, C5b-c9 TCC, C3d, and Factor B also exhibited significant increases, while ZYX, HSP70, and TNF-α showed significant decreases. One study showed no significant changes in IL-6 in AD [33]. The number of miRNA molecules was large, with 14 increasing and 13 decreasing. Additionally, neural cell-derived sEV proteins related to energy metabolism, brain insulin resistance, lysosomes, and oxidative stress exhibited corresponding changes in AD. Among them, Aβ42, p-Tau181, t-Tau, neurogranin, miR-29a-5p, and miR-25b-5p have been studied frequently: Aβ42 protein in 11 studies, p-Tau181 in eight studies, neurogranin in six studies, t-Tau in five studies, miR-29a-5p and miR-125b-5p in four studies each. Compared to HC, levels of Aβ42, Aβ40, t-Tau, p-Tau181, miR-29c-3p, miR-125b-5p, miR-132–5p, miR-210–3p, and C7 in neural cell-derived sEVs are significantly increased in MCI, while neurogranin, synaptophysin, synaptotagmin, synaptotagmin-1, synaptopodin, GAP43, SNAP25, and ZYX are significantly reduced. Therefore, AD and MCI exhibit similar trends in these biomarkers. When comparing AD and MCI groups, neural cell-derived sEV Aβ42, Aβ42/40, t-Tau, p-Tau, p-Tau181, NfL, C4b, Factor D, fragment Bb, C5b-C9 TCC, and miR-29c-3p showed significant increases, while GAP43, neurogranin, SNAP25, and synaptotagmin 1 exhibited significant decreases. Therefore, Aβ42, Aβ42/40, t-Tau, p-Tau, p-Tau181, miR-29c-3p, GAP43, neurogranin, SNAP25, and synaptotagmin can effectively distinguish between AD, MCI, and HC groups. However, as these results are derived from a synthesis of various studies, the specific diagnostic value remains to be further analyzed in subsequent research, which would be a meaningful endeavor.

Table 3 primarily examines whether there are differences in blood glial cell-derived sEVs between AD and HC, focusing on ADsEVs, MDsEVs and ODsEVs. Three studies focused on ADsEVs and found that complement effector proteins such as C1q, C4b, Factor B, Factor D, fragment Bb, C5b, C3b, C3d, and C5b-C9 TCC in patients with AD were increased, while complement regulatory proteins like CD59, DAF, CD46, and CR1 were decreased. Additionally, inflammatory cytokines IL-6, TNF-α, and IL-1β were elevated, BACE-1 and sAPPβ were increased, and GDNF was reduced. One study observed changes in miRNA within the blood glial cell-derived sEVs in patients with AD and found that miR-125b-5p (ADsEVs, MDsEVs, ODsEVs), miR-107 (ADsEVs, MDsEVs, ODsEVs), miR-29a-5p (ADsEVs, ODsEVs), miR-132–5p (ADsEVs, MDsEVs), miR-106b-5p (MDsEVs), miR-210–3p (MDsEVs), and miR-135b-5p (ODsEVs) were increased, while miR-29a-5p (MDsEVs) was decreased.

Two studies compared differences between NDsEVs and ADsEVs in patients with AD in the same indicators [44,46], finding that levels of Aβ42, p-Tau181, p-S396-Tau, sAPPα, sAPPβ, BACE-1, γ-secretase, TNF-α, C5b-C9 TCC, C3d, C4b, Bb, Factor B, and Factor D in ADsEVs were significantly higher than those in NDsEVs. One study found that TNF-α in NDsEVs of patients with AD was lower than in the HC group, but that in ADsEVs it was higher than HC [44]. Another study found that miR-29a-5p in NDsEVs of patients with AD was lower than among HC, but that in ADsEVs it was higher than HC [19]. These conflicting results require further validation due to small sample sizes.

### 3.4 Longitudinal study results

The longitudinal studies mainly observed changes in neural cell-derived sEVs from the early clinical stage of AD (when cognitive function was normal) to AD diagnosis, to identify early predictive biomarkers (Table 5). A total of 11 articles were included, with 10 focusing on NDsEVs and one on ADsEVs. There were two primary observation methods among the longitudinal study. Six studies adopted a prospective design, which involves enrolling healthy subjects initially and following them over time to observe whether they progress to AD after several years; three studies utilized a retrospective design, in which patients with AD were retrospectively analyzed to find blood samples collected years before, when their cognitive function was normal. The remaining two articles did not clearly specify the observation sequence.

**Table 5:** Changes in blood neural-derived sEV protein of patients with AD, compared with preAD, from longitudinal studies.

Number	Author/Publication year	Observation time	Groups (sample size)	Literature quality	Changes in neural cell-derived sEV proteins with time	Results
1	Longfei Jia et al. 2021	5–7 years	longitudinal study-AD1 (normal) (160) longitudinal study-AD2 (AD) (160) longitudinal study-HC1 (normal) (160) longitudinal study-HC2 (normal) (160)	high-quality	GAP43↓, neurogranin↓, SNAP25↓, synaptotagmin 1↓	The plasma NDsEV GAP43, neurogranin, SNAP25 and synaptotagmin 1 were not effective in detecting preclinical AD when used alone. However, the composite model (GAP43+neurogranin+SNAP25+synaptotagmin 1+APOEε4) can be effectively predicted and was an effective biomarker 5–7 years before AD onset.
2	Aonan Zhao et al. 2020	2–3 years	longitudinal study-MCI1 (70) longitudinal study-AD1 (2 years) (8) longitudinal study-MCI2 (62) longitudinal study-AD2 (3 years) (16)	high-quality	Aβ42↑, SS-16 scores↓	With MCI transformed into AD, plasma NDsEV Aβ42 increased and SS-16 score decreased significantly.
3	Dimitrios Kapogiannis et al. 2019	1–10 years	longitudinal study-HC (baseline) (222) longitudinal study-HC (normal) (94) longitudinal study-AD (128)	high-quality	p-Tau231↑, p-Tau181↑, p-S312-IRS-1↑, pY-IRS-1↑, t-Tau (no change), Aβ42 (no change)	Compared with preAD, plasma NDsEV p-Tau181, p-Tau231, p-S312-IRS-1, and pY-IRS-1 increased significantly in patients with AD, but the t-Tau and Aβ42 had no changes.
4	Edward J Goetzl et al. 2018	6–11 years	longitudinal study-HC (18) longitudinal study-AD1 (normal) (18) longitudinal study-AD2 (AD) (18)	high-quality	AMPA4↓, NPTX2↓, NLGN1↓, NRXN2α↓	Compared with preAD, plasma NDsEV AMPA4, NPTX2, NLGN1, and NRXN2α decreased significantly in patients with AD. Compared with HC, plasma NDsEV AMPA4, NLGN1, and NRXN2α decreased significantly in patients with preAD.
5	Edward J Goetzl et al. 2016	1–10 years	longitudinal study-HC (9) longitudinal study-AD1 (normal) (9) longitudinal study-AD2 (AD) (9) longitudinal study-FTC (10) longitudinal study-FTD1 (normal) (10) longitudinal study-FTD2 (FTD) (10)	high-quality	synaptotagmin↓, synaptopodin↓, synaptophysin↓, neurogranin↓, GAP43↓	Compared with HC, plasma NDsEV synaptotagmin, synaptophysin, neurogranin, synaptopodin, and GAP43 decreased significantly in patients with preAD. Compared with preAD, above NDsEVs decreased significantly in patients with AD. Compared with FTC, plasma NDsEV synaptotagmin, synaptophysin, and neurogranin decreased significantly in patients with preFTD, but there were no changes between the preFTD and FTD. Compared with preFTD, plasma NDsEV synaptopodin and GAP43 decreased significantly in patients with FTD, but there were no changes between the preFTD and FTC. Compared with FTD, plasma NDsEV synaptotagmin, synaptopodin, synaptophysin, and GAP43 decreased significantly in patients with AD.
6	Erin L. Abner et al. 2016	3–11 years	longitudinal study-time1 (normal) (18) longitudinal study-time2 (3–11years later) (20)	high-quality	Aβ42↑, p-Tau181↑, Cathepsin D↑, REST↓, neurogranin↓, p-S396-Tau (no change)	With normal aging, plasma NDsEV p-Tau181, Aβ42, cathepsin D, and REST increased, but neurogranin decreased significantly. There was no changes in p-S396-Tau.
7	Dimitrios Kapogiannis et al. 2015	1–10 years	longitudinal study-HC (26) longitudinal study-AD1 (normal) (22) longitudinal study-AD2 (AD) (22)	high-quality	p-panY-IRS-1↓, p-S312-IRS-1↑, p-S312-IRS-1/p-panY-IRS-1↑	Compared with HC, plasma NDsEV p-S312-IRS-1, and p-S312-IRS-1/p-panY-IRS-1 increased, p-panY-IRS-1 decreased significantly in patients with preAD, but there was no changes between preAD and AD with AD development.
8	Edward J Goetzl et al. 2015	1–10 years	longitudinal study-HC (20) longitudinal study-AD1 (normal) (20) longitudinal study-AD2 (AD) (20)	high-quality	cathepsin D↑, LAMP-1↑, ubiquitin↑, HSP70↓	Compared with HC, plasma NDsEV cathepsin D, LAMP-1, and ubiquitin increased, HSP70 decreased significantly in patients with preAD, but there was no changes between preAD and AD with AD development.
9	Edward J. Goetzl et al. 2015	2–10 years	longitudinal study-HC (16) longitudinal study-AD1 (normal) (16) longitudinal study-AD2 (AD) (16)	high-quality	LRP6↓, HSF1↓, REST↓	Compared with HC, plasma NDsEV LRP6, HSF1, and REST decreased significantly in patients with preAD. Compared with preAD, above NDsEVs decreased significantly in patients with AD.
10	Massimo S. Fiandaca et al. 2015	1–10 years	longitudinal study-HC (57) longitudinal study-AD1 (normal) (24) longitudinal study-AD2 (AD) (24)	high-quality	p-S396-Tau↑, p-Tau181↑, Aβ42↑	Compared with HC, blood NDsEV p-S396-Tau, p-Tau181, and Aβ42 increased significantly in patients with preAD. Compared with preAD, above NDsEVs increased significantly in patients with AD.
11	Edward J. Goetzl et al. 2017 △	5–12 years	longitudinal study-HC (16) longitudinal study-AD1 (normal) (16) longitudinal study-AD2 (AD) (16)	high-quality	C4b↑△, C3d↑△, Factor B↑△, Bb↑△, C3b↑△, C5b-C9 TCC↑△, CD59↓△, DAF↓△	Compared with preAD, plasma NDsEV C4b, C3d, Factor B, Bb, C3b, and C5b-C9 TCC increased significantly in patients with AD, but there was no changes between preAD and HC. Compared with HC, plasma NDsEV CD59 and DAF decreased significantly in patients with preAD. Compared with preAD, above NDsEVs decreased significantly in patients with AD.

△: Just detect the ADsEV molecules. After literature quality evaluation, all the above mentioned literatures are of high quality.

AD: Alzheimer's disease; ADsEVs: astrocyte-derived small extracellular vesicles; AMPA4: glua4-containing glutamate; APOE: Apolipoprotein E; CD46: cluster of differentiation 46 protein; CD59: cluster of differentiation 59 protein; DAF: decay-accelerating factor; FTC: frontotemporal dementia control; FTD: frontotemporal dementia; GAP43: growth associated protein 43; HC: healthy control; HSF1: heat shock factor 1; HSP70: heat-shock protein 70; LAMP: lysosome-associated membrane protein; LRP6: lipoprotein receptor-related protein 6; MCI: mild cognitive impairment; NDsEVs: neuron-derived small extracellular vesicles; NLGN1: neuroligin1; NPTX2: neuronal pentraxin 2; NRXN2α: neurexin 2α; preAD: preclinical AD; REST: repressor element 1-silencing transcription factor; SNAP25: ‌synaptosomal-associated protein, 25 kDa; SS-16: sniffin sticks 16; TCC: terminal complement complex.

In the 11 longitudinal studies of NDsEVs, Aß-related proteins, Tau-related proteins, synapse related proteins, immunoinflammatory-related proteins, and miRNAs showed significant changes with AD progression (Table 6). Among the Aß-related proteins, four studies showed that Aβ42 increased, while one study showed no significant change. Among Tau-related proteins, p-Tau181, p-Tau231, and p-S396-Tau increased significantly, while t-Tau and p-S396-Tau showed no significant change in one study. Among synaptic related proteins, neurogranin, GAP43, SNAP25, synaptotagmin 1, AMPA4, NPTX2, NLGN1, NRXN2α, synaptotagmin, synaptopodin, synaptophysin, and neurogranin showed significant decreases. In immunoinflammatory-related proteins, complement effect proteins C4b, C3d, Factor B, Factor D, fragment Bb, C3b, and C5b-c9 TCC increased, while complement regulatory proteins CD59 and DAF decreased, and heat shock protein HSP-70 decreased. Among other proteins, brain insulin resistance-related proteins p-S312-IRS-1, pY-IRS-1, and p-S312-IRS-1/p-panY-IRS-1 increased, p-panY-IRS-1 decreased, lysosome-associated proteins cathepsin D, LAMP-1, and ubiquitin increased, and transcription-related proteins LRP6, HSF1, and REST decreased. These individual markers, used alone or in combination with others, could be used to predict AD progression within 11 years. Among these, Aβ42, p-Tau181, neurogranin, GAP43, p-S312-IRS-1, and cathepsin D were reported in two or more studies. Additionally, some studies indicated that [49,50] in preAD, neural cell-derived sEV cathepsin D, LAMP-1, ubiquitin, p-S312-IRS-1, and p-S312-IRS-1/p-panY-IRS-1 were already elevated, and that HSP70 and p-panY-IRS-1 decreased. However, these indicators did not change significantly with AD development. In one longitudinal study, complement effect proteins C4b, C3d, Factor B, Bb, C3b, and C5b-C9 TCC increased in blood ADsEVs of patients with AD, while complement regulatory proteins CD59 and DAF decreased, consistent with the directional changes observed in NDsEVs.

**Table 6:** Classification of and changes in blood neural cell-derived sEV protein of patients with AD, compared with preAD, from longitudinal studies.

Classification of sEV molecules	Changes in sEV molecules, compared with preAD	sEV molecules
Aß-related proteins	Increase↑	Aβ42(*4)
	No change	Aβ42
Tau-related proteins	Increase↑	p-Tau181(*3), p-Tau231, p-S396-Tau
	No change	t-Tau, p-S396-Tau
Synaptic related proteins	Decrease↓	neurogranin(*3), GAP43(*2), SNAP25, synaptotagmin 1, AMPA4, NPTX2, NLGN1, NRXN2α, synaptotagmin, synaptopodin, synaptophysin, neurogranin
Immunoinflammatory-related proteins	Increase↑	C4b△, C3d△, Factor B△, Bb△, C3b△, C5b-C9 TCC△
	Decrease↓	HSP70, CD59, DAF△
others	Increase↑	Lysosome-related proteins: cathepsin D(*2), LAMP-1, ubiquitin Brain insulin resistance related proteins: p-S312-IRS-1(*2), pY-IRS-1, p-S312-IRS-1/P-panY-IRS-1
	Decrease↓	Brain insulin resistance related protein: p-panY-IRS-1 Transcription-related proteins: LRP6, HSF1, REST

△: Just detect the ADsEV molecules.

Aβ42(*4): There were four studies about Aβ42 herein.

AD: Alzheimer's disease; ADsEVs: astrocyte-derived small extracellular vesicles; AMPA4: glua4-containing glutamate; CD59: cluster of differentiation 59 protein; DAF: decay-accelerating factor; GAP43: growth associated protein 43; HSF1: heat shock factor 1; HSP70: heat-shock protein 70; LAMP: lysosome-associated membrane protein; LRP6: lipoprotein receptor-related protein 6; NLGN1: neuroligin1; NPTX2: neuronal pentraxin 2; NRXN2α: neurexin 2α; PreAD: preclinical AD; REST: repressor element 1-silencing transcription factor; SNAP25: ‌synaptosomal-associated protein, 25 kDa; TCC: terminal complement complex;.

### 3.5 Clinical diagnostic value of findings

Among the 34 articles, 22 validated the early diagnostic value of neural cell-derived sEV proteins for AD through receiver-operating characteristic (ROC) curve analysis, three examined the early predictive value of neural cell-derived sEVs, and 12 conducted correlation analyses of the relations between neural cell-derived sEV proteins and cognitive function (Table 7). Based on the area under the ROC curve (AUC), this study summarizes sEV biomarkers with high diagnostic value (AUC > 0.85) and moderate diagnostic value (0.7 ≤ AUC < 0.85) in AD. Table 8 provides a summary of sEV molecules with high diagnostic and early predictive value (AUC > 0.85) in AD.

**Table 7:** ROC curve analyses of blood neural cell-derived sEV molecules in patients with AD and correlations with cognitive function.

Number	Author/Publication year/Literature quality/Groups (sample size)	Changes in sEVs	Analytical method	sEV molecules	AUC/p/r	Sensibility	Specificity	Conclusion
1	Ashish Kumar et al. 2023. high-quality HC (11) MCI (11) MCI-AD (6) AD (11)	miR–9–5p↑, miR-106b-5p↑, miR-125b-5p↑, miR–132–5p↑, miR-29a-5p↓	ROC curve (NDsEVs)	miR-29a-5p, miR-125b-5p and miR–210–3p (predict global cognitive function alone)	AUC=0.948	—	—	Plasma NDsEV MiR-106b-5p can be used as a new blood biomarker of AD.
				miR–210–3p, miR–132–5p (predict MCI alone)	AUC=0.941	—	—	
				miR-106b-5p (AD vs HC)	AUC=1.000	—	—	
			correlation analysis (NDsEVs)	miR-106b-5p and the thickness of temporal lobe cortex	p<0.005	—	—	There was a significant negative correlation between plasma NDsEV miR-106b-5p and the thickness of temporal lobe cortex in patients with AD.
		miR-29a-5p↑, miR-125b-5p↑, miR–132–5p↑, miR-107↑	ROC curve (ADsEVs)	miR-107 and miR–210–3p (predict global cognitive function alone)	AUC=0.964	—	—	Plasma ADsEV miR-107 can be used as a new blood biomarker of AD.
				miR-107 and miR–210–3p (predict MCI alone)	AUC=0.941	—	—	
				miR-107 (AD vs HC)	AUC=1.000	—	—	
			correlation analysis (ADsEVs)	miR-107, miR–132–5p and the thickness of temporal lobe cortex	p<0.05	—	—	There was a significant negative correlation between plasma ADsEV miR-107, miR–132–5p and the thickness of temporal lobe cortex in patients with AD.
		miR-29a-5p↓, miR-125b-5p↑, miR–132–5p↑, miR-107↑, miR–210–3p↑	ROC curve (MDsEVs)	miR-29a-5p, miR-106b-5p (predict global cognitive function alone)	AUC=0.925	—	—	Plasma MDsEV miR–132–5p and miR-125b-5p can be used as new blood biomarkers of AD.
				miR-29a-5p (predict MCI)	AUC=0.840	—	—	
				miR–132–5p, miR-125b-5p (AD vs HC)	AUC=1.000	—	—	
			correlation analysis (MDsEVs)	miR-106b-5p, miR–132–5p and the thickness of temporal lobe cortex	p<0.05	—	—	There was a significant negative correlation between plasma MDsEV miR-106b-5p, miR–132–5p and the thickness of temporal lobe cortex in patients with AD.
		miR-29a-5p↑, miR-107↑, miR-135b-5p↑, miR-125b-5p↑	ROC curve (ODsEVs)	miR-125b-5p (predict global cognitive function)	AUC=0.753	—	—	Plasma ODsEV miR-29a-5p can be used as a new blood biomarker of AD.
				miR-29a-5p (AD vs HC)	AUC=1.000	—	—	
			correlation analysis (ODsEVs)	miR-29a-5p and the thickness of temporal lobe cortex	p<0.05	—	—	There was a significant negative correlation between plasma ODsEV miR-29a-5p and the thickness of temporal lobe cortex in patients with AD.
2	Chen Tian et al. 2022. high-quality discovery-AD1 (45) discovery-PD (84) discovery-HC1 (32) validation-AD2 (66) validation-HC2 (82)	NMDAR2A↓, L1CAM↓, Aβ40↓, Aβ42↓, p-Tau231↓, p-Tau396↓	ROC curve	discovery cohort-NMDAR2A (AD vs HC)	AUC=0.890	0.820	0.810	Blood NDsEV NMDAR2A and L1CAM carrying synaptic function- and brain-related proteins were potential biomarkers for AD.
				discovery cohort-LICAM (AD vs HC)	AUC=0.779	0.510	0.910	
				discovery cohort-NMDAR2A+LICAM+Aβ42+Aβ40+*p*-Tau231+*p*-Tau396 (AD vs HC)	AUC=0.915	0.850	0.840	
				validation cohort-NMDAR2A (AD vs HC)	AUC=0.809	0.740	0.670	
				validation cohort-LICAM (AD vs HC)	AUC=0.762	0.720	0.700	
				validation cohort-NMDAR2A+LICAM+Aβ42+Aβ40+*p*-Tau231+*p*-Tau396 (AD vs HC)	AUC=0.810	0.810	0.630	
3	Devrim Yagmur Durur et al. 2022. moderate-quality discovery-AD (8) discovery-HC (8) validation-AD (20) validation-HC (15)	let-7e-5p↑, miR–96–5p↑, miR-484↑, miR-99b-5p↓, miR–100–5p↓, miR-30e-5p↓, miR-378i↓, miR–145–5p↓, miR-378c↓, miR-451a↓	ROC curve	let-7e-5p (AD vs HC)	AUC=0.921	0.857	1.000	Plasma NDsEV let-7e was a potential biomarker for AD.
4	Erden Eren et al. 2022. moderate-quality pure AD (21) mixed AD (40)	Aβ42, p-Tau181, t-Tau, synaptophysin, synaptopodin (no changes)	correlation analysis	Aβ42 and different aspects of cognitive function	—	—	—	Higher NDsEV Aβ42 levels were consistently associated with better cognitive status, memory, fluency, working memory and executive function. Higher NDsEV p-Tau181 levels were associated with worse digit span backward performance and worse TMT-B scores. Both NDsEV t-Tau/Aβ42 and p-Tau/Aβ42 ratios were associated with better forward digit span, but p-Tau/Aβ42 was associated with worse backward digit span. Higher levels of NDEV synaptic integrity-related biomarkers (synaptophysin and synaptopodin) were associated with better performance on executive function tasks.
				p-Tau181 and backward digit span, p-Tau181 and TMT-B (executive function)		—	—	
				t-Tau/Aβ42, p-Tau/Aβ42 and forward digit span		—	—	
				p-Tau/Aβ42 and backward digit span		—	—	
				synaptophysin and TMT-B (executive function)		—	—	
				synaptopodin and TMT-B (executive function)		—	—	
5	Tao‑Ran Li et al. 2022. high-quality HC (Aβ-) (84) HC (Aβ+) (72) MCI (45) AD (45)	Aβ40↑, Aβ42↑	ROC curve	Aβ42 (AD vs HCAβ-)	AUC=0.957	—	—	Plasma NDsEV Aβ42 was a diagnostic biomarker of AD, which can distinguish Aβ+ HC from Aβ- HC.
				Aβ42 (MCI vs HCAβ-)	AUC=0.857	—	—	
				Aβ42+APOE ε4 (HCAβ+ vs HCAβ-)	AUC=0.705	—	—	
			correlation analysis	Aβ42 and entorhinal cortex	p<0.05	—	—	Plasma NDsEV Aβ42 was related to the volume of the olfactory cortex in HC(Aβ+).
				Aβ42 and MMSE	*r*=−0.454	—	—	Plasma NDsEV Aβ42 was negatively correlated with MMSE.
				Aβ42 and MoCA	*r*=−0.474	—	—	Plasma NDsEV Aβ42 was negatively correlated with MoCA.
				Aβ42 and the changes of MMSE scores among 14 months	*r*=−0.361	—	—	Plasma NDsEV Aβ42 is negatively correlated with the change of MMSE in longitudinal study.
				Aβ42 and the changes of MoCA scores among 14 months	*r*=−0.364	—	—	Plasma NDsEV Aβ42 is negatively correlated with the change of MoCA in longitudinal study.
6	X Anton Alvarez et al. 2022. high-quality HC (20) AD (116)	Aβ42↑, t-Tau↑, p-Tau181↑ (AD) p-S396-Tau↑ (mild to moderate AD) neurogranin↓, REST↓ (mild to moderate AD)	correlation analysis	NDsEV Aβ42, p-Tau181 and serum BDNF	—	—	—	There was a significant negative correlation between plasma NDsEV Aβ42 and serum BDNF, plasma NDsEV p-Tau181 and serum BDNF in patients with mild to moderate AD. There was a positive correlation between plasma NDsEV t-Tau and plasma TNF-α.
				NDsEV Tau and plasma TNF-α	—	—	—	
7	Ying Li et al. 2022. high-quality SCD (76) MCI (80) AD (80) VD (40) HC (40)	Aβ42↑, Aβ42/40↑, t-Tau↑, p-Tau181↑, NfL↑, miR-29c-3p↑	ROC curve	Aβ42/40 (MCI vs HC)	AUC=0.884	—	—	Plasma NDsEV Aβ42/40 and miR-29c-3p can be used as new blood biomarkers of AD.
				Aβ42/40 (AD vs HC)	AUC=0.969	—	—	
				Aβ42/40 (AD vs HC)	AUC=0.806	—	—	
				miR-29c-3p (MCI vs HC)	AUC=0.915	—	—	
				miR-29c-3p (AD vs HC)	AUC=0.906	—	—	
				miR-29c-3p (AD vs MCI)	AUC=0.670	—	—	
				Compound model with Aβ42, Aβ42/40, t-Tau, p-Tau181, NfL, and miR-29c-3p (AD vs HC. AD vs MCI)	—	—	—	The diagnostic value of the composite models was slightly higher than that of the above single NDsEV.
8	Burak Ibrahim Arioz et al. 2021. high-quality HC (23) AD (20)	Alpha-globin↑, Beta-globin↑, Delta-globin↑	ROC curve	hemoglobin (AD vs HC)	AUC=0.691	—	—	Plasma NDsEV hemoglobin might be a diagnosticbiomarker of AD.
9	Longfei Jia et al. 2021. high-quality discovery-AD (28) discovery-MCI (25) discovery-HC (29) validation-AD (73) validation-MCI (71) validation-HC (72)	GAP43↓, neurogranin↓, SNAP25↓, synaptotagmin 1↓	ROC curve (cross-sectional study)	NDsEVs-GAP43 (AD vs HC)	AUC=0.830	—	—	Plasma NDsEV GAP43, neurogranin, SNAP25, and synaptotagmin 1 were a potential biomarker for AD.
				CSF-GAP43 (AD vs HC)	AUC=0.900	—	—	
				CSF-GAP43 (MCI vs HC)	AUC=0.910	—	—	
				NDsEVs-neurogranin (AD vs HC)	AUC=1.000	—	—	
				NDsEVs-neurogranin (AD vs MCI)	AUC=1.000	—	—	
				NDsEVs-neurogranin (MCI vs HC)	AUC=0.820	—	—	
				CSF-neurogranin (AD vs HC)	AUC=0.940	—	—	
				CSF-neurogranin (MCI vs HC)	AUC=0.950	—	—	
				NDsEVs-SNAP25 (AD vs HC)	AUC=0.750	—	—	
				CSF-SNAP25 (AD vs HC)	AUC=0.740	—	—	
				CSF-SNAP25 (MCI vs HC)	AUC=0.730	—	—	
				NDsEVs-synaptotagmin 1 (AD vs HC)	AUC=0.950	—	—	
				NDsEVs-synaptotagmin 1 (AD vs MCI)	AUC=0.760	—	—	
				NDsEVs-synaptotagmin 1 (MCI vs HC)	AUC=0.820	—	—	
				CSF-synaptotagmin 1 (AD vs HC)	AUC=0.800	—	—	
				CSF-synaptotagmin 1 (MCI vs HC)	AUC=0.770	—	—	
				composite models (FAD vs FAD-HC)	AUC=0.870	—	—	This model was used to verify the model in longitudinal study.
			ROC curve (longitudinal study)	composite models (training and test datasets)	AUC=0.880	—	—	Plasma NDsEV GAP43, neurogranin, SNAP25, and synaptotagmin 1 act as effective biomarkers for prediction of AD 5 to 7 years before cognitive impairment.
				composite models (training datasets)	AUC=0.890	—	—	
				composite models (test datasets)	AUC=0.890	—	—	
			correlation analysis	NDsEVs and MMSE	p<0.0001	—	—	There was a significant correlation between MMSE score and plasma NDsEV GAP43, neurogranin, SNAP25 and synaptotagmin 1, which can be used to predict cognitive decline.
			correlation analysis	NDsEVs and CSF	p<0.0001	—	—	Plasma NDsEV GAP43, neurogranin, SNAP25, and synaptotagmin 1 were highly negatively correlated with their expression in CSF, suggesting that these biomarkers may reflect pathological changes in the brain and can be used to help diagnose AD.
10	Aonan Zhao et al. 2020. high-quality HC (80) MCI (87) AD (88)	Aβ42↑, SS-16 scores↓	ROC curve (longitudinal study)	2-year follow-up: SS-16 scores (AD vs MCI)	AUC=0.810	0.750	0.742	The combination of plasma NDsEV Aβ42 and SS-16 scores more accurately predicted the transformation from MCI to AD.
				2-year follow-up: Aβ42 (AD vs MCI)	AUC=0.840	0.875	0.614	
				2-year follow-up: Aβ42+SS-16 scores	AUC=0.930	0.875	0.871	
				3-year follow-up: SS-16 scores (AD vs MCI)	AUC=0.830	0.625	0.774	
				3-year follow-up: Aβ42 (AD vs MCI)	AUC=0.840	0.875	0.614	
				3-year follow-up: Aβ42+SS-16 scores (AD vs MCI)	AUC=0.950	0.938	0.855	
			ROC curve (cross-sectional study)	Aβ42 (MCI vs HC)	AUC=0.690	—	—	Plasma NDsEV Aβ42, SS-16 scores and their combination have high diagnostic value for AD.
				Aβ42 (AD vs HC)	AUC=0.900	—	—	
				SS-16 (MCI vs HC)	AUC=0.650	—	—	
				SS-16 (AD vs HC)	AUC=0.900	—	—	
				Aβ42+SS-16 (MCI vs HC)	AUC=0.710	—	—	
				Aβ42+SS-16 (AD vs HC)	AUC=0.960	—	—	
11	Dongmei Gu et al. 2020. high-quality AD (31) HC (15)	MMP-9↑, Aβ42↑, p-Tau181↑, p-S396-Tau (no change), IL-6 (no change)	ROC curve	Aβ42 (AD vs HC)	AUC=0.710	0.774	0.600	Plasma NDsEV MMP‐9 might be a potential biomarker for AD.
				p-Tau181 (AD vs HC)	AUC=0.660	0.807	0.553	
				MMP-9 (AD vs HC)	AUC=0.800	0.613	0.933	
			correlation analysis	NDsEVs and MMSE	—	—	—	There was no correlation between Aβ42, p-Tau181, MMP-9 and MMSE in patients with AD.
12	Eunjoo Nam et al. 2020. moderate-quality HC (26) MCI (30) AD (20)	p-tau↑, t-Tau↑	correlation analysis	p-Tau (S202) and MMSE	*p* = 0.05	—	—	There was a negative correlation between serum NDsEV p-Tau and MMSE, and a positive correlation between serum p-Tau/t-Tau and MMSE in patients with AD. Serum NDsEV tau protein (t-Tau and p-Tau) was a useful biomarker for monitoring the progress of AD.
				p-Tau (S202)/t-Tau and MMSE	p<0.05	—	—	
			ROC curve	t-Tau>30.94 pg/mL (AD vs HC)	AUC=0.643	0.437	0.882	
				p-Tau>11.85 pg/mL (AD vs HC)	AUC=0.730	0.467	1.000	
				p-Tau (S202)/t-Tau=0.39 (AD vs HC)	AUC=0.611	0.938	0.438	
13	Nan Zhang et al. 2020. high-quality HC (15) AD (24)	TDP-43↑	correlation analysis	TDP-43 and cognitive function, TDP-43 and neuropsychiatric symptoms, TDP-43 and APOE genotype	p>0.100	—	—	There was no correlation between plasma NDsEV TDP-43 and cognitive function, neuropsychiatric symptoms or APOE genotype in patients with AD.
14	Charisse N. Winston et al. 2019. moderate-quality HC (20) MCI (20) MCI-AD (20) AD (20)	C1q↑, C4b↑, Factor D↑, Factor Bb↑, C5b↑, C3b↑, C5b-C9 TCC↑, DAF↓, CD46↓, CD59↓, CR1↓	ROC curve	Bb, C3b, C1q, C4b, C5b, TCC, Factor D, DAF, CD46, CD59, CR1 individually (MCI-AD vs MCI, AD vs HC)	—	√	—	Plasma ADsEV complement protein was a component of neurotoxic neuritis and may be a biomarker for MCI to transform into AD.
				C4b, Bb, CD59 individually (AD vs MCI-AD)	—	√	—	
				Bb, C3b, C4b, DAF, CD46 individually (AD vs HC, AD vs MCI-AD, MCI-AD vs MCI, MCI vs HC)	—	√	—	
15	Cristina Agliardi et al. 2019. moderate-quality HC (17) AD (24)	SNAP25↓	ROC curve	SNAP25 (AD vs HC)	AUC=0.826	0.875	0.706	Serum NDsEV SNAP-25 might be an effective biomarker of AD, which can reflect the integrity of synapses in the brain.
			correlation analysis	SNAP25 and MMSE	*r* = 0.465	—	—	There was a positive correlation between serum NDsEV SNAP25 and MMSE.
16	Diana J Cha et al. 2019. moderate-quality HC (16) MCI (16) AD (31)	miR–132–3p↓, miR–212–3p↓	ROC curve	miR-132 (AD vs HC)	AUC=0.770	—	—	Plasma NDsEV miR-132 and miR-212 could distinguish AD from HC, miR-212 could distinguish AD from HC, HC from MCI. Plasma NDsEV miR-132 and miR-212 might be potential diagnostic biomarkers for AD.
				miR-212 (AD vs HC)	AUC=0.840	—	—	
				miR-212 (AD and MCI)	AUC=0.680	—	—	
17	Dimitrios Kapogiannis et al. 2019. high-quality HC (35) AD (29)	p-Tau231↑, p-Tau181↑, p-S312-IRS-1↑, pY-IRS-1↑, t-Tau (no change), Aβ42 (no change)	ROC curve (longitudinal study)	age+gender+sample type+NDsEV concentration+NDsEV mean diameter+*t*-Tau+*p*-Tau181+*p*-S312-IRS-1+pY-IRS-1 (BLSA cohort-training datasets)	AUC=0.896	0.818	0.858	This composite model had a good predictive value for 9 years before AD onset, and Aβ42 had no contribution to the final model.
				age+gender+sample type+NDsEV concentration+NDsEV mean diameter+*t*-Tau+*p*-Tau181+*p*-S312-IRS-1+pY-IRS-1 (BLSA cohort-test datasets)	AUC=0.800	0.556	0.887	
			ROC curve (cross-sectional study)	age+gender+sample type+NDsEV concentration+NDsEV mean diameter+*t*-Tau+*p*-Tau181+*p*-S312-IRS-1+pY-IRS-1 (JHADRC cohort-training datasets)	AUC=0.989	1.000	0.947	This composite model had a good diagnostic value in distinguishing AD from HC.
				age+gender+sample type+NDsEV concentration+NDsEV mean diameter+*t*-Tau+*p*-Tau181+*p*-S312-IRS-1+pY-IRS-1 (JHADRC cohort-test datasets)	AUC=0.767	0.917	0.600	
			correlation analysis	p-Tau181 and cognitive function	p<0.05	—	—	Higher p-Tau181 was associated with worse verbal memory, attention, executive function, and visuospatial function cross-sectionally.
				p-S312-IRS-1 and cognitive function	p<0.05	—	—	Higher p-S312-IRS-1 was associated with worse verbal memory and executive function.
18	Longfei Jia et al. 2019. moderate-quality discovery-AD (28) discovery-MCI (25) discovery-HC (29) validation-AD (73) validation-MCI (71) validation-HC (72)	Aβ42↑, t-Tau↑, p-Tau181↑	correlation analysis	Aβ42 and CSF Aβ42	p<0.0001	—	—	There was a negative correlation between plasma NDsEV Aβ42 and CSF Aβ42, while there was a positive correlation between plasma NDsEV t-Tau, p-Tau181 and CSF t-Tau, p-Tau181.
				t-Tau and CSF t-Tau	p<0.0001	—	—	
				p-Tau181 and CSF p-Tau181	p<0.0001	—	—	
			ROC curve	NDsEVs-Aβ42 (AD vs HC)	AUC=0.930	—	—	Plasma NDsEV Aβ42, t-Tau and p-Tau181 could distinguish AD from HC, AD from MCI, MCI from HC when used alone or in combination.
				NDsEVs-Aβ42 (AD vs MCI)	AUC=0.830	—	—	
				NDsEVs-Aβ42 (MCI vs HC)	AUC=0.740	—	—	
				NDsEVs-t-Tau (AD vs HC)	AUC=0.890	—	—	
				NDsEVs-t-Tau (AD vs MCI)	AUC=0.720	—	—	
				NDsEVs-t-Tau (MCI vs HC)	AUC=0.790	—	—	
				NDsEVs-p-Tau181 (AD vs HC)	AUC=0.880	—	—	
				NDsEVs-p-Tau181 (AD vs MCI)	AUC=0.760	—	—	
				NDsEVs-p-Tau181 (MCI vs HC)	AUC=0.730	—	—	
				CSF-Aβ42 (AD vs HC)	AUC=0.930	—	—	
				CSF-Aβ42 (AD vs MCI)	AUC=0.820	—	—	
				CSF-Aβ42 (MCI vs HC)	AUC=0.760	—	—	
				CSF-t-Tau (AD vs HC)	AUC=0.880	—	—	
				CSF-t-Tau (AD vs MCI)	AUC=0.700	—	—	
				CSF-t-Tau (MCI vs HC)	AUC=0.780	—	—	
				CSF-p-Tau181 (AD vs HC)	AUC=0.900	—	—	
				CSF-p-Tau181 (AD vs MCI)	AUC=0.760	—	—	
				CSF-p-Tau181 (MCI vs HC)	AUC=0.720	—	—	
				NDsEVs-Aβ42+*t*-Tau+*p*-Tau181 (AD vs HC)	AUC=0.980	—	—	
				NDsEVs-Aβ42+*t*-Tau+*p*-Tau181 (AD vs MCI)	AUC=0.880	—	—	
				NDsEVs-Aβ42+*t*-Tau+*p*-Tau181 (MCI vs HC)	AUC=0.850	—	—	
				CSF-Aβ42+*t*-Tau+*p*-Tau181 (AD vs HC)	AUC=0.980	—	—	
				CSF-Aβ42+*t*-Tau+*p*-Tau181 (AD vs MCI)	AUC=0.890	—	—	
				CSF-Aβ42+*t*-Tau+*p*-Tau181 (MCI vs HC)	AUC=0.860	—	—	
19	Charisse N Winston et al. 2018. moderate-quality HC (76) MCI (61)	Aβ42↑, neurogranin↓, synaptophysin↓, synaptotagmin↓, synaptopodin↓	ROC curve	Aβ42, neurogranin, synaptophysin, synaptotagmin, synaptopodin individually (MCI and HC)	—	—	—	Plasma NDsEV Aβ42, neurogranin, synaptophysin, synaptotagmin, and synaptopodin could accurately distinguish MCI from HC.
20	Edward J Goetzl et al. 2018. high-quality HC (28) AD (28)	AMPA4↓, NPTX2↓, NLGN1↓, NRXN2α↓	correlation analysis	AMPA4 and the extent of cognitive loss	*r*=−0.559	—	—	The decrease of plasma NDsEV AMPA4 and NLGN1 was negatively correlated with the extent of cognitive loss, while was positively correlated with the MMSE.
				NLGN1 and the extent of cognitive loss	*r*=−0.667	—	—	
				AMPA4 and MMSE	*r* = 0.621	—	—	
				NLGN1 and MMSE	*r* = 0.525	—	—	
21	Edward J Goetzl et al. 2016. high-quality AD (12) HC (12) FTD (16) FTC (16)	BACE-1↑, sAPPβ↑, GDNF↓	ROC curve	BACE-1, sAPPβ (AD vs HC)	—	—	—	Plasma ADsEV BACE-1 and sAPPβ could distinguish AD from HC.
22	Charisse N Winston et al. 2016. moderate-quality-quality HC (10) MCI (20) MCI-AD (20) AD (10)	Aβ42 ↑ p-Tau181 ↑ p-S396-Tau ↑ neurogranin ↓ REST ↓	ROC curve	Aβ42 (AD与HC)	AUC=0.980	—	—	Plasma NDsEV Aβ42, p-Tau181, p-S396-Tau, neurogranin, and REST could accurately distinguish AD from HC.
				p-Tau181 (AD与HC)	AUC=1.000	—	—	
				p-S396-Tau (AD与HC)	AUC=0.980	—	—	
				neurogranin (AD与HC)	AUC=1.000	—	—	
				REST (AD与HC)	AUC=1.000	—	—	
				Aβ42 (AD与MCI)	AUC=0.980	—	—	Plasma NDsEV Aβ42, p-S396-Tau, neurogranin, and REST could accurately distinguish AD from MCI.
				p-Tau181 (AD与MCI)	AUC=0.693	—	—	
				p-S396-Tau (AD与MCI)	AUC=0.980	—	—	
				neurogranin (AD与MCI)	AUC=1.000	—	—	
				REST (AD与MCI)	AUC=1.000	—	—	
				Aβ42 (MCI与HC)	AUC=0.765	—	—	Plasma NDsEV p-Tau181, neurogranin, and REST could accurately distinguish MCI from HC.
				p-Tau181 (MCI与HC)	AUC=0.875	—	—	
				p-S396-Tau (MCI与HC)	AUC=0.783	—	—	
				neurogranin (MCI与HC)	AUC=0.973	—	—	
				REST (MCI与HC)	AUC=1.000	—	—	
23	Edward J Goetzl et al. 2016. high-quality AD (12) HC (12) FTD (16) FTC (16)	synaptotagmin↓, synaptopodin↓, synaptophysin↓, neurogranin↓, GAP43↓	ROC curve	synaptophysin (AD vs HC)	AUC=1.000	—	—	The levels of all plasma NDsEV synaptic protein synaptophysin, synaptotagmin, synaptopodin, GAP43, and neurogranin decreased significantly, which might be an early biomarker of AD.
				synaptotagmin (AD vs HC)	AUC=0.990	—	—	
				synaptopodin (AD vs HC)	AUC=0.970	—	—	
				GAP43 (AD vs HC)	AUC=0.790	—	—	
				neurogranin (AD vs HC)	AUC=0.990	—	—	
			correlation analysis	synaptopodin and MMSE and ADAS-cog	*r*=−0.80	—	—	There was a negative correlation between plasma NDsEV synaptopodin, synaptophysin and ADAS-cog, while there was a positive correlation between plasma NDsEV synaptopodin, synaptophysin and MMSE.
				synaptophysin and MMSE and ADAS-cog	*r*=−0.73	—	—	
24	Dimitrios Kapogiannis et al. 2015. high-quality AD (26) HC (26) FTD (16) FTC (16) DM2 (20) DC (20)	p-panY-IRS-1↓, p-S312-IRS-1↑, p-S312-IRS-1/p-panY-IRS-1↑	ROC curve	IRS-1 (AD vs HC)	AUC=0.703	—	—	Plasma NDsEV p-S312-IRS-1 could distinguish AD from HC.
				p-S312-IRS-1 (AD vs HC)	AUC=0.932	—	—	
				p-panY-IRS-1 (AD vs HC)	AUC=1.000	—	—	
				p-panY-IRS-1 (DM2 and DC)	AUC=0.741	—	—	
				p-S312-IRS-1 (DM2 and DC)	AUC=0.999	—	—	
				p-S312-IRS-1 (FTD and FTC)	AUC=0.928	—	—	
25	Edward J Goetzl et al. 2015. high-quality AD (26) HC (26) FTD (16) FTC (16)	cathepsin D↑, LAMP-1↑, ubiquitin↑, HSP70↓	ROC curve	cathepsin D (AD vs HC)	AUC=1.000	—	—	Blood NDsEV cathepsin D could distinguish AD from HC.
26	Massimo S. Fiandaca et al. 2015. high-quality AD (57) HC (57) FTD (16) FTC (16)	t-Tau↑, p-S396-Tau↑, p-Tau181↑, Aβ42↑	ROC curve	p-Tau181+*p*-S396-Tau+Aβ42+*t*-Tau (AD vs HC)	AUC=0.999	—	—	Blood NDsEV Aβ42, p-S396-Tau, and p-Tau181 could distinguish AD from HC.
				p-Tau181 (AD vs HC)	AUC=0.991	—	—	
				p-S396-Tau (AD vs HC)	AUC=0.988	—	—	
				Aβ42 (AD vs HC)	AUC=0.987	—	—	
				t-Tau (AD vs HC)	AUC=0.731	—	—	

*r* < 0.3: no correlation; 0.3 ≤ *r* < 0.5: slight correlation; 0.5 ≤ *r* < 0.8: moderate correlation; *r* ≥ 0.8: highly correlation; 0.5<AUC<0.7: low diagnostic value; 0.7≤AUC<0.85: moderate diagnostic value; AUC≥0.85: high diagnostic value.

AD: Alzheimer's disease; ADsEVs: astrocyte-derived small extracellular vesicles; AMPA4: glua4-containing glutamate; APOE: Apolipoprotein E; AUC: area under the curve; BACE-1: β-site amyloid precursor protein-cleaving enzyme 1; CR1: complement receptor type 1; CSF: cerebrospinal fluid; DAF: decay-accelerating factor; FAD: amilial Alzheimer's disease; GAP43: growth associated protein 43; GDNF: glial-derived neurotrophic factor; HSP70: heat-shock protein 70; IL-6: interleukin-6; L1CAM: L1 cell adhesion molecule; LAMP: lysosome-associated membrane protein; MDsEVs: microglia-derived small extracellular vesicles; MMP-9: matrix metalloproteinase-9; NDsEVs: neuron-derived small extracellular vesicles; NfL: neurofilament light chain; NLGN1: neuroligin1; NMDAR2A: N-methyl-d-aspartate receptor 2A; NPTX2: neuronal pentraxin 2; NRXN2α: neurexin 2α; ODsEVs: oligodendrocyte-derived small extracellular vesicles; REST: repressor element 1-silencing transcription factor; ROC: receiver-operating characteristic; sAPP: soluble amyloid precursor protein; SNAP25: ‌synaptosomal-associated protein, 25 kDa; SS-16: sniffin sticks 16; TCC: terminal complement complex; TDP-43: TAR DNA binding protein of 43 kDa.

**Table 8:** Blood neural cell-derived sEVs with high diagnostic and early predictive value for AD (AUC>0.85).

PART 1: sEV molecules with high diagnostic value for AD in cross-sectional studies (AUC>0.85)		
Classification of sEV molecules	Changes of sEV molecules	sEV molecules
Aß-related proteins	Increase↑	Aβ42(*5), Aβ42/40
Tau-related proteins	Increase↑	p-Tau181(*3), p-S396-Tau(*2), t-Tau
Synaptic related proteins	Decrease↓	neurogranin(*3), synaptotagmin, synaptotagmin1, synaptopodin, synaptophysin, NMDAR2A
miRNAs	Increase↑	miR-29c-3p
Others	Increase↑	p-S312-IRS-1, cathepsin D
	Decrease↓	REST

PART 2: sEV molecules (used in combination) with high diagnostic value for AD in cross-sectional studies (AUC>0.85)	
sEV molecules (used in combination)	AUC
NMDAR2A+LICAM+Aβ42+Aβ40+*p*-Tau231+*p*-Tau396	0.915
Aβ42+Aβ42/40+*t*-Tau+*p*-Tau181+NfL+miR-29c-3p	>0.969
age+gender+sample type+NDsEV concentration+NDsEV mean diameter +*t*-Tau+*p*-Tau181+*p*-S312-IRS-1+pY-IRS-1	0.989
Aβ42+*t*-Tau+*p*-Tau181	0.980
p-Tau181+*p*-S396-Tau+Aβ42+*t*-Tau	0.999

PART 3: sEV molecules with high early predictive value for AD in longitudinal studies (AUC>0.85)	
Composite model	Function of the model
Aβ42 + SS-16 scores	This model could predict the transformation from MCI to AD in 2–3 years.
age+gender+sample type+NDsEV concentration+NDsEV mean diameter+*t*-Tau+*p*-Tau181+*p*-S312-IRS-1+pY-IRS-1.	This model had a good predictive value for 9 years before AD onset.
GAP43+neurogranin+SNAP25+synaptotagmin1+APOEε4	This model had a good predictive value for 5–7 years before AD onset.

△: ADsEVs; □: MDsEVs; ☆: ODsEVs; unmarked: NDsEVs.

Aβ42(*4): There were four studies about Aβ42 herein.

AD: Alzheimer's disease; ADsEVs: astrocyte-derived small extracellular vesicles; APOE: Apolipoprotein E; AUC: area under the curve; GAP43: growth associated protein 43; L1CAM: L1 cell adhesion molecule; MCI: mild cognitive impairment; MDsEVs: microglia-derived small extracellular vesicles; NDsEVs: neuron-derived small extracellular vesicles; NMDAR2A: N-methyl-d-aspartate receptor 2A; ODsEVs: oligodendrocyte-derived small extracellular vesicles; SNAP25: synaptosomal-associated protein, 25 kDa; SS-16: sniffin sticks 16.

In cross-sectional studies, ROC curves revealed that compared with HC, Aβ- and Tau-related proteins (Aβ42, Aβ42/40, BACE-1, sAPPβ, t-Tau, p-Tau181, and p-S396-Tau), synaptic related proteins (neurogranin, synaptophysin, synaptotagmin, synaptopodin, GAP43, SNAP-25, NMDAR2A, and L1CAM), complement proteins (Bb, C3b, C1q, C4b, C5b, TCC, Factor D, DAF, CD46, CD59, and CR1), miRNAs (miR-29c-3p, miR-29a-5p, miR-106b-5p, miR-107, miR-125b-5p, miR-132, miR-132–5p, miR-212, and let-7e-5p), other proteins (MMP-9, p-S312-IRS-1, pY-IRS-1, p-panY-IRS-1, cathepsin D, REST, and hemoglobin) had moderate or higher diagnostic value in AD when used individually (area under the curve [AUC] ≥70%). Among these, Aβ- and Tau-related proteins (Aβ42, Aβ42/40, p-S396-Tau, and p-Tau181), synaptic related proteins (neurogranin, synaptotagmin, synaptophysin, synaptopodin, and NMDAR2A), miRNA molecules miR-106–5p(NDsEVs), miR-29c-3p(NDsEVs), let-7e-5p(NDsEVs), miR-107 (ADsEVs), miR-132–5p (MDsEVs), miR-125b-5p (MDsEVs), miR-29a-5p (ODsEVs), and other proteins (p-S312-IRS-1, cathepsin D, and REST) had individual diagnostic AUC exceeding 85%, indicating high diagnostic value as a biomarker. When Aβ42, t-Tau, and p-Tau181 were used in combination, the AUC was 0.980. Interestingly, five studies found that the AUC was higher when multiple indicators were used together compared to the use of a single indicator alone (Table 8, Part 2). However, due to the lack of external validation and the fact that each indicator exhibited high diagnostic value, it remains unclear which individual indicator or combination of indicators is superior. Comparing AD to MCI, neurogranin showed high diagnostic value, while synaptotagmin showed moderate diagnostic value. Comparing MCI to HC, Aβ42, Aβ42/40, miR-29c-3p, miR-107 (ADsEVs), and miR-132–5p (MDsEVs) showed high diagnostic value, and neurogranin, synaptotagmin, miR-210–3p (NDsEVs), miR-132–5p (NDsEVs), and miR-29a-5p (ODsEVs) showed moderate diagnostic value. Therefore, neurogranin and synaptotagmin in neural cell-derived sEVs can effectively distinguish between the three groups of AD, MCI and HC (Table 9).

**Table 9:** Reviewed CSF-related indexes in patients with AD.

Number	Author/Publication year/Literature quality	Groups	Diagnostic criteria	Changes in neural cell-derived sEVs	CSF index	Role of CSF	Correlation analysis between CSF and neural cell-derived sEVs
1	Chen Tian et al. 2022. high-quality	discovery cohort-AD1 (45) discovery cohort-PD (84) discovery cohort-HC1 (32) validation cohort-AD2 (66) validation cohort-HC2 (82)	t-Tau/Aβ42 in CSF meets the AD standard, Aβ42=880 pg/ml, p-Tau/Aβ42=0.028, t-Tau/Aβ42=0.33	NMDAR2A↓, L1CAM↓, Aβ40, Aβ42↓, p-Tau231, p-Tau396↓	t-Tau/Aβ42	T-Tau/Aβ42 in CSF was used as an objective indicator in AD diagnosis.	—
2	Ying Li et al. 2022. high-quality	SCD (76) MCI (80) AD (80) VD (40) HC (40)	NIA-AA criteria	Aβ42↑, Aβ42/40↑, t-Tau↑, p-Tau181↑, NfL↑, miR-29c-3p↑	Aβ42, p-Tau181, miR-29c-3p	These indexes in CSF were used for correlation analysis.	There was a high negative correlation between Aβ42 in plasma NDsEV and in CSF, while there was a high positive correlation between t-Tau, p-Tau181, miR-29c-3p in plasma NDsEV and in CSF. There was no correlation between NfL in plasma NDsEV and in CSF.
3	Longfei Jia et al. 2021. high-quality	discovery-AD (28) discovery-MCI (25) discovery-HC (29) validation-AD (73) validation-MCI (71) validation-HC (72) longitudinal study-AD1 (normal) (160) longitudinal study-AD2 (AD) (160) longitudinal study-HC1 (normal) (160) longitudinal study-HC2 (normal) (160)	MMSE, MoCA, CDR, NIA-AA criteria	GAP43↓, neurogranin↓, SNAP25↓, synaptotagmin 1↓	Aβ42, p-Tau, t-Tau, GAP43, neurogranin, SNAP25, synaptotagmin 1	P-Tau/Aβ42 and t-Tau/Aβ42 in CSF were used as an objective indicator in AD diagnosis. GAP43, neurogranin, SNAP25 and synaptotagmin 1 in CSF were used for correlation analysis.	There was a high negative correlation between GAP43, neurogranin, SNAP25, synaptotagmin 1 in plasma NDsEV and in CSF.
4	Pamela J. Yao et al. 2021. moderate-quality	AD1 (22) HC1 (29) AD2 (14) HC2 (14)	NIA-AA criteria, IWG-2 criteria, Aβ42<192 pg/ml and p-Tau181>23 pg/ml in CSF	ATP synthase↓, SOD1↓, complex I (1)↓, complex I (6)↓, complex III↓, complex IV↓	Aβ42, p-Tau181	These indexes in CSF were only used as diagnostic criteria.	—
5	Maria Serpente et al. 2020. high-quality	AD (40) HC (40)	Aβ42<600 pg/ml, tau>500 pg/ml (>70 years old), 450 pg/ml (50–70 years old), p-Tau181>61 pg/ml in CSF	miR-23a-3p↑, miR–223–3p↑, miR-190a-5p↑, miR–100–3p↓,	Aβ, t-Tau, P-Tau181	These indexes in CSF were only used as diagnostic criteria.	—
6	Charisse N. Winston et al. 2019. moderate-quality	HC (20) MCI (20) MCI-AD (20) AD (20)	Aβ42<192 pg/ml in CSF, IWG-2 criteria, CDR=0.5–1.0, MMSE	C1q↑, C4b↑, Factor D↑, Bb↑, C5b↑, C3b↑, C5b-C9 TCC↑, DAF↓, CD46↓, CD59↓, CR1↓	Aβ42	These indexes in CSF were only used as diagnostic criteria.	—
7	Longfei Jia et al. 2019. moderate-quality	discovery-AD (28) discovery-MCI (25) discovery-HC (29) validation-AD (73) validation-MCI (71) validation-HC (72)	NIA-AA criteria	Aβ42↑, t-Tau↑, p-Tau181↑	Aβ, t-Tau, p-Tau181	These indexes in CSF were used for correlation analysis.	There was a high negative correlation between Aβ42 in plasma NDsEV and in CSF, while there was a high positive correlation between t-Tau, p-Tau181 in plasma NDsEV and in CSF.
8	Edward J Goetzl et al. 2018. high-quality	HC (28) AD (28) longitudinal study-HC (18) longitudinal study-AD1 (normal) (18) longitudinal study-AD2 (AD) (18)	Aβ42<192 pg/ml and p-Tau181>23 pg/ml in CSF, NIA criteria, IWG-2 criteria	AMPA4↓, NPTX2↓, NLGN1↓, NRXN2α↓	Aβ42, p-Tau181	These indexes in CSF were only used as diagnostic criteria.	—
9	Edward J. Goetzl et al. 2017. high-quality	HC (28) AD (28) longitudinal study-HC (16) longitudinal study-AD1 (normal) (16) longitudinal study-AD2 (AD) (16)	Aβ42<192 pg/ml, p-Tau181>23 pg/ml in CSF, NIA-AA criteria, IWG-2 criteria, MMSE, ADAS-cog, CDR=0.5–1.0	C1q↑, C4b↑, C3d↑, Factor B↑, Factor D↑, Bb↑, C3b↑, C5b-C9 TCC↑, IL-6↑, TNF-α↑, IL-1β↑, CD59↓, DAF↓, CD46↓, CR1↓	Aβ42, p-Tau181	These indexes in CSF were only used as diagnostic criteria.	—
10	Edward J Goetzl et al. 2016. high-quality	AD (12) HC (10) FTD (14) FTC (10)	Aβ42<192 pg/ml in CSF, CDR, MMSE, ADAS-cog	BACE-1↑, sAPPβ↑, GDNF↓	Aβ42, t-Tau, p-Tau181	These indexes in CSF were only used as diagnostic criteria.	—
11	Erin L. Abner et al. 2016. moderate-quality	AD (10) HC (10) longitudinal study-time1 (normal) (18) longitudinal study-time2 (3–11years later) (20)	ADAS-cog, neurological examination, psychological test, CSF protein, MRI	Aβ42↑, p-Tau181↑, p-S396-Tau↑cathepsin D↑, REST↓, neurogranin↓	Aβ42	These indexes in CSF were only used as diagnostic criteria.	—
12	Dimitrios Kapogiannis et al. 2015. high-quality	AD (26) HC (26) FTD (16) FTC (16) DM2 (20) DC (20) longitudinal study-AD1(normal) (22) longitudinal study-AD2(AD) (22)	Aβ42<192 pg/ml in CSF, CDR, MMSE, ADAS-cog	p-panY-IRS-1↓, p-S312-IRS-1↑, p-S312-IRS-1/p-panY-IRS-1↑	Aβ42, t-Tau, p-Tau181	Aβ42 in CSF were used as an objective indicator in AD diagnosis. Aβ42, t-Tau and p-Tau181 in CSF were used for correlation analysis.	There was no significant correlation between p-panY-IRS-1, p-S312-IRS-1, p-S312-IRS-1/p-panY-IRS-1 in plasma NDsEVs and t-Tau, p-Tau181 in CSF individually. But after controlling for gender and age, there was a weak negative correlation between p-S312-IRS-1 in plasma NDsEVs and Aβ42 in CSF.
13	Edward J Goetzl et al. 2015. high-quality	AD (26) HC (26) FTD (16) FTC (16) longitudinal study-HC (20) longitudinal study-AD1(normal) (20) longitudinal study-AD2(AD) (20)	Aβ42<192 pg/ml in CSF, NIA criteria, NINCDS-ADRDA criteria, CDR, MMSE	cathepsin D↑, LAMP-1↑, ubiquitin↑, HSP70↓	Aβ42	These indexes in CSF were only used as diagnostic criteria.	—

AD: Alzheimer's disease; ADAS-cog: Alzheimer's disease assessment scale-cognitive subscale; AMPA4: glua4-containing glutamate; ATP: adenosine triphosphate; BACE-1: β-site amyloid precursor protein-cleaving enzyme 1; CD46: cluster of differentiation 46 protein; CD59: cluster of differentiation 59 protein; CDR: clinical dementia rating; CR1: complement receptor type 1; CSF: cerebrospinal fluid; DAF: decay-accelerating factor; DM2: type 2 diabetes mellitus; FTC: frontotemporal dementia control; FTD: frontotemporal dementia; GAP43: growth associated protein 43; GDNF: glial-derived neurotrophic factor; HC: healthy control; HSP70: heat-shock protein 70; IL-1β: interleukin-1β; IL-6: interleukin-6; IWG: international working group; L1CAM: L1 cell adhesion molecule; LAMP: lysosome-associated membrane protein; MCI: mild cognitive impairment; MMSE: mini mental state examination scale; MoCA: montreal cognitive assessmen; MRI: magnetic resonance imaging; NIA-AA: National Institute on Aging-Alzheimer's Association; NDsEVs neuron-derived small extracellular vesicles; NfL: neurofilament light chain; NINCDS-ADRDA: national institute of neurological and communicative disorders and stroke; NLGN1: neuroligin1; NMDAR2A: N-methyl-d-aspartate receptor 2A; NPTX2: neuronal pentraxin 2; NRXN2α: neurexin 2α; PD: Parkinson's disease; REST: repressor element 1-silencing transcription factor; sAPP: soluble amyloid precursor protein; SCD: subjective cognitive decline; SNAP25: ‌synaptosomal-associated protein, 25 kDa; SOD1: ‌superoxide dismutase 1; TCC: terminal complement complex; TNF-α: tumor necrosis factor-α; VD: vascular dementia.

Among the longitudinal studies comparing preAD, ROC curves revealed that three composite models had high predictive value for early AD prediction within 1–10 years. Model 1 (within 2–3 years before AD onset): Aβ42+SS-16 score. Model 2 (within 1–10 years): age+gender+sample type+NDsEV concentration+NDsEV mean diameter+*t*-Tau+*p*-Tau181+*p*-S312-IRS-1+pY-IRS-1. Model 3 (within 5–7 years): GAP43+neurogranin+SNAP25+synaptotagmin 1+APOEε4.

### 3.6 Correlation analyses between neural cell-derived sEVs and CSF molecules

Among the 34 articles included herein, 13 studies collected CSF from patients, and 4 studies analyzed the correlation between the related indexes in CSF and the corresponding markers in neural cell-derived sEVs. One study found negative correlations between the blood neural cell-derived sEVs GAP43, neurogranin, SNAP25, synaptotagmin 1, and their CSF expressions [30]. Two studies [27,41] found a strong negative correlation between Aβ42 in blood neural cell-derived sEVs and in CSF, while t-Tau, p-Tau181, and miR-29c-3p levels in neural cell-derived sEVs showed a strong positive correlation with their levels in CSF, and NfL showed no correlation. One study found that plasma neural cell-derived sEV IRS-1 and p-S312-IRS-1/p-panY-IRS-1 had no significant correlation with t-Tau and p-Tau181 in CSF, but after controlling for gender and age, p-S312-IRS-1 showed a weak negative correlation with Aβ42 in CSF [49]. The other nine articles only used Aβ42, t-Tau, or p-Tau181 in CSF as the main diagnostic basis for patients with AD and did not conduct correlation analysis.

### 3.7 Bias analysis

This article identifies two potential sources of bias. First, the sample sizes across individual studies vary significantly, ranging from a minimum of 29 cases to a maximum of 739 cases, with an average sample size of 165 cases. However, during data analysis, articles with sample sizes less than 60 were only included in the results section without being considered in the final conclusions. Second, in cross-sectional studies, 41.2% of the literature was classified as moderate-quality. However, systematic analysis and summarization revealed that the biomarkers involved in these moderate-quality studies either lacked ROC curve validation or were corroborated by higher-quality studies, thereby not affecting the final conclusions.

## 4 Discussion

The development of AD has experienced an insidious and long process. Clinically, the diagnosis of AD mainly relies on blood biomarkers "A/T/N/I," however, due to the invasiveness and high cost of the examination, they are not suitable for routine screening. Furthermore, "A/T/N/I" biomarkers do not directly reflect changes in neurons within the brain. As diagnostic markers of diseases, blood sEVs are hot topics in clinical research. The sEVs secreted by central nervous system cells can penetrate the blood-brain barrier and reach peripheral blood through blood circulation. Because sEVs carry biological information of parent cells, they can represent the parent cells’ disease characteristics, not only the accumulation of pathological products such as Aβ and Tau protein, but also reveal the early changes of nerve cells themselves. Therefore, detecting the neural cell-derived sEVs in blood has great potential for disease diagnosis and prediction, and is a biomarker with great application prospects.

The extraction methods of clinical blood sEVs include kits and ultracentrifugation. Thirty-three studies included herein extracted sEVs by kits, and only one study used ultracentrifugation [21]. The results showed that these two methods of extraction yielded completely opposite results. It is believed that extraction of sEVs with kits and then extraction of specific tissue or cell-derived sEVs by immunoprecipitation method is the most suitable current technology for large-scale, micro-volume and rapid extraction [53], and blood samples of clinical patients are most commonly used. Although ultracentrifugation is relatively simple, it may lead to particle aggregation and precipitation, potentially lowering sEV concentration during the process of supernatant extraction. Additionally, repeated centrifugation may disrupt the structure of sEVs, resulting in a lower concentration and poorer quality of the extracted protein [54,55], which may explain why the content of sEVs obtained by ultracentrifugation are inconsistent with results using kits. Therefore, the results involved herein were all based on the sEVs extracted by the kits. Neural cells mainly include neurons, astrocytes, microglia and oligodendrocytes. In this paper, after blood sEVs were extracted, neural cell-derived sEVs were extracted by immunoprecipitation, NDsEVs were obtained by targeting neuron marker L1CAM, ADsEVs were obtained by targeting astrocyte marker GLAST, MDsEVs were obtained by targeting microglia marker TMEM119, and ODsEVs were obtained by targeting oligodendrocyte marker PDGFRα. Through analysis and induction, the blood neural cell-derived sEV molecules described herein mainly included Aβ- and Tau-related proteins, synaptic related proteins, immunoinflammatory-related proteins, and miRNAs. The following was a classified discussion, and the results herein were compared with the same molecular content in different body fluids and brain tissues to clarify the characteristics of blood neural cell-derived sEVs, which provided a basis for the clinical application of biomarkers from different sources.

### 4.1 Diagnostic value of Aβ- and Tau-related proteins in cross-sectional studies

Aβ is produced from APP through β-secretase or γ-secretase. Abnormal deposition and aggregation of Aβ in neurons can lead to neuronal damage and death, which is one of the main pathological mechanisms of AD [6]. Aβ42 and Aβ40 primarily differ in the number of amino acid residues, being composed of peptide chains with 42 and 40 amino acids, respectively. Compared with Aβ40, Aβ42 more easily aggregates and forms plaques, resulting in neurotoxic effects, while Aβ40 mainly accumulates around neurons in the extracellular space and vascular walls [56]. Herein, except for the research by ultracentrifugation [21], the remaining nine relevant studies indicated a significant increase in blood neural cell-derived sEV Aβ42 and Aβ42/40 in patients with AD. When used individually, both exhibited a high diagnostic value, consistent with the traditional pathological mechanism of AD. Clinical trials and animal experiments revealed a significant increase in brain tissue Aβ42 and Aβ42/40 [[57], [58], [59], [60]], indicating that changes in blood neural cell-derived sEVs can represent alterations of Aβ in brain tissue. However, Aβ42 and Aβ42/40 changes in CSF are opposite those in neural cell-derived sEVs (Appendix 4). An analysis of 13 studies found a significant decrease in Aβ42 and Aβ42/40 levels in the CSF of patients with AD, while Aβ40 showed no significant change [61], consistent with other research results [62,63]. The studies included herein demonstrated a strong negative correlation between neural cell-derived sEV Aβ42 and CSF Aβ42 in patients with AD, indicating an equivalent diagnostic efficacy [27,41]. Similarly, a significant decrease in Aβ42/40 was observed in plasma of patients with AD [[64], [65], [66], [67], [68]]. Another studies found a marked increase in APP/Aβ42 and Aβ40/Aβ42 (not Aβ42/40) in plasma, consistent with the results of brain Aβ-PET, suggesting a high diagnostic value [69]. These findings indicate a decrease in plasma Aβ42 and Aβ42/40 in AD. In conclusion, the levels of Aβ42 in blood neural cell-derived sEVs and brain tissue of patients with AD were increased, while the levels of Aβ42 in CSF and blood were decreased. The phenomenon is likely related to the metabolism of amyloid-β (Aβ). It is possible that under physiological conditions, Aβ can cross the blood-brain barrier and enter the systemic circulation, where it can be detected in the blood [70]. In the pathological state of AD, Aβ is widely deposited, forming plaques and adhering to neurons, which are not easily shed into CSF and blood, so the content of Aβ in CSF and blood of patients with AD decreases [71]. Therefore, although the decrease of Aβ42 and Aβ42/40 in blood and CSF is of diagnostic significance, it is opposite to the direction of brain tissue change. While blood neural cell-derived sEVs are small vesicles secreted by neural cells, which reflect the pathological changes of neurons themselves and are consistent with the direction of brain tissue change.

Tau protein, a microtubule-associated protein participating in the formation of nerve cytoskeleton, mainly exists in axons of neurons and helps to maintain the morphological structure and function of neurons [72]. However, excessive phosphorylation transforms tau protein from soluble to an insoluble state, leading to pathological aggregation, promoting the formation of NfT, and causing loss of neuronal function and cytotoxicity [73], which is another major pathological mechanism of AD [74]. Increased concentrations of p-Tau181 and p-S396-Tau, representing the phosphorylation status of serine residues at positions 181 and 396 on tau protein, are closely associated with the pathological characteristics of AD. In this study, p-Tau181 and p-S396-Tau in plasma neural cell-derived sEVs were significantly increased, and individually exhibited high AD diagnostic value, indicating that 181 and 396 are the main phosphorylation sites of tau protein in AD. Their high phosphorylation levels cause tau protein to lose its normal binding ability to microtubules, affecting the neuronal cytoskeleton structure and function, leading to abnormal neuronal conduction and causing cognitive impairment. Similarly, p-Tau181 [65,[75], [76], [77], [78]] and p-S396-Tau [79] were significantly elevated in the CSF and plasma of patients with AD, and p-Tau181 in plasma reached a peak 4–8 years before patient death and stabilized thereafter, indicating its potential prognostic predictive value [80]. This suggests that changes of p-Tau181 and p-S396-Tau in blood neural cell-derived sEVs, CSF, and blood are consistent (Appendix 4). A animal experiment has found that injecting p-S396-Tau, the plasma neural cell-derived sEVs of patients with AD, into the brain of mice can promote the pathological deposition of p-Tau [73]. Furthermore, studies included herein found that the combined use of neural cell-derived sEVs Aβ42, p-Tau181, and t-Tau exhibited higher diagnostic value [41].

### 4.2 Diagnostic value of synapse proteins in cross-sectional studies

Synapse is the normal connection between neurons and a key part of nerve information transmission, which plays a vital role in learning, memory and cognition of nervous system [81]. Related studies have shown that AD has significant synaptic plasticity disorder, that is, the structure and function of synapses change, and such changes already exist in the early stage of AD [82,83]. Synaptic proteins are the basis of information transmission between synapses, and play an important role in regulating neurotransmitter release and participating in early neuronal development [84]. Synaptic protein locations are divided into presynaptic membrane protein and postsynaptic membrane protein. Presynaptic membrane proteins can promote the fusion of presynaptic membranes with synaptic vesicles, playing a role in neurotransmitter release [85,86]. Postsynaptic membrane proteins are primarily involved in neurotransmitter reception and the transmission of neuronal signaling molecules [87], thus the quantity and functional state of presynaptic and postsynaptic membrane proteins play a crucial role in the interneuronal information transmission.

This systematic review indicates that synaptic proteins in blood neural cell-derived sEVs of patients with AD are significantly reduced, aligning with the pathological mechanisms of AD. Among these proteins, neurogranin, synaptotagmin (synaptotagmin 1), synaptophysin, synaptopodin, and NMDAR2A have high diagnostic value when used alone. Among them, synaptotagmin (synaptotagmin 1) and synaptophysin are presynaptic membrane proteins. Synaptotagmin is primarily involved in neurotransmitter release and synaptic plasticity, among which synaptotagmin 1 is one, associated with synaptic vesicle exocytosis activities. Synaptophysin is predominantly found in synaptic vesicles, playing a crucial role in their formation and neurotransmitter release. In contrast, neurogranin, synaptopodin and NMDAR2A are postsynaptic membrane proteins. Neurogranin participates in the regulation of calcium signaling and synaptic plasticity in presynaptic neurons, associated with the processes of learning and memory. Synaptopodin regulates the morphology and function of postsynaptic neurons, participates in the formation of postsynaptic protein complexes, regulates the stability of postsynaptic proteins, and plays a role in synaptic plasticity. NMDAR2A, a subunit of the NMDA-type glutamate receptor mainly involves in synaptic plasticity and learning and memory, also participates in the regulation of calcium channels, affecting the electrophysiological activity of postsynaptic membranes. The results herein showed that these five synaptic proteins in blood neural cell-derived sEVs of patients with AD decreased significantly, indicating that the contents of both presynaptic and postsynaptic membrane proteins in the brain neurons were significantly decreased. Meanwhile, compared with HC (Section 3.3 of Results), MCI already had significant reductions in the synaptic proteins neurogranin, synaptophysin, synaptotagmin (synaptotagmin-1), synaptopodin, GAP43 and SNAP25. The decrease of synaptic proteins can lead to structural dysfunction of neurons related to synaptic plasticity disorder, poor release and reception of neurotransmitters, abnormal ion channels and electrical activity, and eventually impair learning and memory function, resulting in cognitive dysfunction.

Studies have revealed distinct directional changes in the synaptic proteins of patients with AD from different sources, including blood neural cell-derived sEVs, brain tissue, CSF, and blood (Appendix 4). Herein, six studies investigated synaptic proteins in blood neural cell-derived sEVs, all of which demonstrated a significant decrease in all 13 synaptic proteins. This aligns with the changes observed in synaptic protein content in brain tissue from other studies, in which neurogranin [88], synaptotagmin [89], synaptopodin [90], and synaptophysin [[91], [92], [93]–94] were significantly reduced in the brain tissue of both patients with AD and AD model mice, while NMDAR2A levels decreased [95]. These results suggest that synaptic proteins in blood neural exosomes indeed reflect changes in brain tissue. However, the changes in synaptic protein in CSF are opposite to those observed in blood neural cell-derived sEVs and brain tissue. Studies have indicated a significant increase in neurogranin [[96], [97], [98]] and synaptotagmin [99,100] in the CSF of patients with AD, both of which hold AD diagnostic value. One study included herein demonstrated a decrease in synaptotagmin 1 in blood neural cell-derived sEVs of patients with AD, which was negatively correlated with the content in CSF [30]. It also suggests that changes in synaptic proteins in CSF do not represent real changes in neurons themselves. The opposite results between plasma neural cell-derived sEVs and CSF may be related to the blood-CSF barrier, or to the different sources of synaptic proteins, but the specific mechanism needs to be further studied. As blood neural cell-derived sEVs reflect changes in brain tissue itself, their results are consistent with the changes in brain tissue, while studies have shown that neurogranin in the blood of patients with AD is unchanged [98,101], which may be related to the dilution of synaptic proteins after they are introduced into peripheral blood, and the specific reasons remain to be further confirmed. These above also shows that compared with blood, synaptic proteins in blood neural cell-derived sEVs can reflect the pathological changes of brain tissue itself, which not only has high diagnostic value for AD, but more importantly, can show significant changes in the early clinical stage, which has early diagnostic value.

### 4.3 Diagnostic value of immunoinflammatory-related proteins in cross-sectional studies

The relations between immune inflammation and AD have attracted increasing attention. Research showed that pathological immune responses and excessive inflammation can accelerate aging and are closely associated with AD [102]. Immune inflammatory reaction can induce the activation of M1 microglia, leading to excessive production of inflammatory factors, triggering diffuse neuronal damage, eventually resulting in cognitive impairments [103,104]. The complement system is an important component of the body's immune system, involved in regulating inflammation, clearing pathogens and apoptotic cells, and regulating autoimmune responses. Complement system proteins can mediate the activation of A1-type astrocytes (neurotoxic) and M1-type microglia (pro-inflammatory) to promote neuronal damage, and can also compensate for the lack of clearance of Aβ in AD and trigger related adaptive immune responses [105,106]. Complement system proteins mainly include complement effector proteins and complement regulatory proteins. Complement effector proteins directly participate in complement activation, promoting immune responses and pathogen clearance, while complement regulatory proteins participate in regulating the activity of the complement system, preventing excessive inflammatory responses and self-tissue damage, and protecting host cells from complement attack.

The literature included herein showed a significant increase in complement effector proteins (Bb, C3b, C1q, C4b, C5b, C5b-C9 TCC, and Factor D) and a significant decrease in complement regulatory proteins (DAF, CD46, CD59, and CR1) in plasma NDsEVs and ADsEVs of patients with AD, which had moderate diagnostic value when used independently. It indicated that the immune response of neurons and astrocytes increased in patients with AD. Previous research showed that in AD mouse models, the complement effector protein C1q can promote the activation of C3 (including C3a and C3b) and microglia, leading to the release of inflammatory factors and cytotoxins and the damage of neurons and synapses, resulting in synapse losses [107]. C3b may also promote the generation of C5b-C9 TCC, directly causing neuronal damage [44]. Another study found that the application of complement effector protein antagonists in AD mouse models significantly improved memory and reduce pathological markers such as Aβ, indicating that the overactivation of complement effector proteins accelerates inflammatory responses and promote the development of AD [108]. The absence of complement regulatory protein CD59 has been found to promote cell lysis, significantly associated with synaptic loss, while overexpression of CD59 has a protective effect on cells, preventing such lysis [109]. CR1 helps clear amyloid plaques, and the reduction of CR1 is closely related to various cognitive domains [110]. These processes demonstrate the involvement of immune inflammatory responses in the pathological processes of AD, but the diagnostic value of the literature included herein has yet to be verified through clinical ROC curve analysis and requires further confirmation.

### 4.4 Diagnostic value of miRNAs in cross-sectional studies

miRNAs are noncoding single-stranded RNA molecules approximately 22 nucleotides in length encoded by endogenous genes, which are involved in the expression and regulation of post-transcriptional genes, including the differentiation of neurons, the regulation of plasticity, the proliferation and apoptosis of cells and the regulation of immune metabolism. Different miRNAs have been found to exhibit varying changes in AD [111] and are closely related to synaptic function and specific signals during memory development [112]. A meta-analysis including 770 patients with AD and 664 HC showed that miRNAs have high sensitivity, specificity, and diagnostic odds ratio in diagnosing AD [113].

In the cross-sectional studies included herein, neural cell-derived sEV miRNAs are mainly derived from neurons and glial cells. Neurons are the basic units of the nervous system, which are mainly responsible for receiving, processing, and transmitting information. Glial cells, the supporting cells of the nervous system, include astrocytes, microglia, and oligodendrocytes; they are mainly responsible for supporting, nourishing, protecting, and repairing neurons, and participating in synaptic regulation. Herein, miR-29c-3p (NDsEVs), miR-106b-5p (NDsEVs), let-7e-5p (NDsEVs), miR-107 (ADsEVs), miR-132–5p (MDsEVs), miR-125b-5p (MDsEVs), and miR-29a-5p (ODsEVs) were found to be significantly increased in patients with AD and have high diagnostic values. However, previous studies have found that these miRNAs have a protective effect on neurons. For example, miR-106b-5p can promote cell proliferation and inhibit apoptosis by targeting thioredoxin and thioredoxin‐interacting protein (TXNIP), exerting a neuroprotective effect on SH-SY5Y cells (a type of nerve cell) [114]. Cell experiments have shown that inhibiting miR-132–5p can block BDNF-induced dendrite formation [115]. The animal study showed that the reduction of miR-29a-5p levels can exacerbate neuronal damage by promoting the polarization of M1 microglia [116]. Through combining site prediction and dual luciferase reporter gene experiments, it was found that miR-29c-3p [117,118], miR-29a-5p [119], miR-107 [120], miR-125b-5p [121] can directly target and inhibit BACE 1, having potential anti-AD effects. These studies suggest that elevated levels of sEV miRNAs in the blood may represent a compensatory protective response.

The direction of miRNA changes in blood neural cell-derived sEVs and brain tissue of patients with AD differ significantly (Appendix 4). miRNAs with high diagnostic value in blood neural cell-derived sEVs are significantly elevated; except for miR-125b, which is increased in brain tissue [122], the other six miRNAs are significantly decreased in brain tissue of patients with AD, opposite to the changes in blood neural cell-derived sEVs [117,119,120,[123], [124], [125], [126], [127]]. Blood neural cell-derived sEVs theoretically reflect changes in brain tissue, but the opposite results of miRNA may be related to the secretion mechanism of sEVs. The formation and secretion process of sEVs includes endocytosis, the formation of multivesicular bodies, and fusion with the cell membrane, ultimately releasing into the extracellular environment [128]. This process plays a crucial role in regulating miRNA loading, protection, and expression, thereby significantly impacting miRNA changes. For example, endocytosis and exocytosis may affect the efficiency of miRNA loading, while the binding of miRNA to specific protein complexes (such as HSP90) may further regulate its distribution and stability within vesicles [129]. The double-layer lipid membrane structure of exosomes protects miRNA from degradation by intracellular RNA degrading enzymes; however, miRNA in brain tissue may lack this double-layer membrane protection and thus be susceptible to degradation by RNA degrading enzymes. Additionally, oxidative stress and cellular metabolic states during exosome secretion may influence miRNA expression patterns and levels [130]. These factors could all contribute to the observed changes in miRNA. After consulting the large body of previous research, these seven miRNAs in CSF and blood of patients with AD were found to increase and decrease, with no obvious regularity [121,[131], [132], [133], [134], [135], [136], [137]]. These conflicting results reflect the limitations and nonspecificity of current miRNA diagnostic research. It is known that one miRNA can regulate multiple mRNAs, and one mRNA is regulated by multiple miRNAs, forming a complex network of miRNA-mRNA interactions. Thus their specificity and clinical value for diagnosis require further consideration. Additionally, AD development is a dynamic, continuous process [138], and different stages of miRNAs may exhibit differences. In addition, miRNAs are related to the pathogenesis of various neurodegenerative diseases, which may be another reason for differing results [139]. Therefore, future work should validate the specificity of miRNAs in non-AD neurodegenerative diseases such as vascular dementia and Parkinson's disease.

### 4.5 Predictive value of longitudinal cohort studies

In longitudinal cohort studies, three composite models have shown high predictive value in the early stages of AD (within 1–10 years before onset).

Model 1: Aβ42+SS-16 score. The SS-16 score reflects olfactory discrimination ability, and studies have found Aβ deposits in the olfactory bulb or other brain areas related to olfactory function [140,141]. Impairment in odor identification is closely associated with AD onset [142,143]. ROC curve analysis from longitudinal studies revealed that Aβ42 has a moderate predictive value for AD. Interestingly, when combined with the SS-16 score, the AUC for predicting AD within 2–3 years increased from 0.84 to 0.95, indicating that the combined use of both factors has significant clinical value for predicting the progression from MCI to AD.

Model 2: age+gender+sample type+NDsEV concentration+NDsEV mean diameter+*t*-Tau+*p*-Tau181+*p*-S312-IRS-1+pY-IRS-1. T-Tau represents the total tau protein, including normal and abnormally phosphorylated forms, and is crucial for maintaining neuronal structure and function. Studies have shown that phosphorylated tau is formed in brain tissue before appearance of AD symptoms [144,145], and that elevation of t-Tau in plasma is significantly correlated with AD [63]. A study published in Nature Medicine showed that plasma p-Tau181 is significantly increased in preAD, is further increased in MCI and AD with disease development, and it is highly similar to CSF p-Tau181 in differentiating neurodegenerative diseases from AD and non-AD [146]. The p-Tau181 in CSF and plasma is an effective predictor of longitudinal Tau accumulation, even in the early stage of Tau deposition [147]. Excessive phosphorylation of tau protein can induce brain insulin resistance [148]. The pY-IRS-1 and p-S312-IRS-1 are phosphorylated forms of insulin receptor substrate-1 (IRS-1) at specific sites, and both participate in the insulin signal transduction pathway. Brain insulin resistance weakens the response of brain neurons to insulin, resulting in brain glucose metabolism disorder, which plays an important role in the pathogenesis of AD. This model emphasizes the importance of pY-IRS-1 and p-S312-IRS-1 as predictive biomarkers in AD, and are thus among the model factors with high predictive value.

Model 3: GAP43+neurogranin+SNAP25+synaptotagmin 1+APOEε4. GAP43 is a presynaptic membrane protein involved in the growth of axons, synaptic plasticity, and regulation of learning and memory functions. It plays a crucial role in neuronal development and synapse formation, serving as an important mediator for neuronal survival and plasticity [149]. SNAP-25, another presynaptic membrane protein, not only controls exo/endocytic processes at the presynaptic terminal, but also regulates postsynaptic receptor trafficking and plasticity. Defects of SNAP-25 may contribute to psychiatric diseases by impacting not only presynaptic but also postsynaptic functions [150]. APOEε4, the ε4 allele of apolipoprotein E, is among the significant genetic risk factors for AD and can induce excessive phosphorylation of tau protein, degeneration of GABAergic neurons, and accelerate the deposition of Aβ and tau proteins [151,152]. Neurogranin and synaptotagmin 1 are both presynaptic membrane proteins involved in synaptic transmission. The combined use of these five synaptic proteins could greatly improve the predictive value, with AUC reaching 0.89, which was great significant for discovering the ultra-early changes of AD.

In addition to the above three prediction models, Aβ42, p-Tau181, neurogranin, GAP43, p-S312-IRS-1 and cathepsin D in blood neural cell-derived sEVs were confirmed to predict the occurrence of AD by more than two studies respectively. Therefore, these six proteins holded greater early predictive value for AD. Cathepsin D is related to the pathogenesis of AD and involved in the processing of APP and Tau protein [153]. Studies have found that cathepsin D is highly expressed in brain tissue and low expression in plasma of AD after death [154]. Changes of blood neural cell-derived sEVs can thus help predict the likelihood of patients developing AD in the preAD stages (normal cognitive function or MCI). This provides a valuable time window for the early prevention and treatment of AD, holding significant clinical value.

## 5 Conclusions

This article systematically summarizes the diagnostic and predictive value of blood neural cell-derived sEVs in AD, including 34 studies with a total of 5601 participants. In cross-sectional studies, there are 15 proteins with high diagnostic value, mainly including Aβ and tau-related proteins, synaptic-related proteins, and miRNAs. Specifically, compared with HC, Aβ and tau-related proteins (Aβ42, Aβ42/40, p-S396-tau, p-Tau181) increased, synaptic-related proteins (neurogranin, synaptotagmin, synaptotagmin 1, synaptopodin, synaptophysin, NMDAR2A) decreased significantly in patients with AD. In addition, miR-29c-3p, cathepsin D and p-S312-IRS-1 were significantly increased, while REST was decreased. In longitudinal studies, three composite models containing Aβ, tau, or synaptic-related proteins demonstrate high predictive value before the onset of AD. Furthermore, six proteins that have been studied more than twice and possess predictive value are identified: elevated levels of Aβ42, p-Tau181, and cathepsin D; decreased levels of neurogranin and GAP43; and elevated levels of p-S312-IRS-1. These three predictive models and six key proteins exhibit high early predictive value 1–10 years before the onset of AD.

The detection of blood neural cell-derived sEVs is convenient in clinical practice, with the characteristics of accurate diagnosis and efficient prediction. Compared with the existing blood clinical markers, the synaptic proteins in blood neural cell-derived sEVs are a new class of clinical diagnostic markers. Blood neural cell-derived sEV Aβ and tau-related proteins, synaptic proteins and miRNAs can not only accurately diagnose AD, but more importantly, predict the occurrence of AD 1–10 years in advance. At the same time, by comparing the change trend of diagnostic markers from different sources, it is found that blood neural cell-derived sEVs can better reflect the pathological changes of neurons in brain tissue than blood and CSF, and detect not only the pathological products related to AD, but also the change rules of neurosynaptic proteins. This systematic review provides important evidence for the diagnosis and early prediction of AD, finds new diagnostic markers and early prediction value, and highlights the advantages of blood neural cell-derived sEVs.

## 6 Advantages, limitations and prospects

This paper has systematically reviewed and summarized the diagnostic and predictive value of blood neural cell-derived sEVs in AD in the published literature for the first time, presenting obvious advantages. Fortunately, blood neural cell-derived sEVs, carrying biological information from parent cells, can reflect the changes in proteins or genes in brain tissue and be easily detectable in blood, presenting good application value. To date, 34 published studies worldwide have explored the diagnostic value of blood neural cell-derived sEVs in AD. However, a systematic review and compilation of that literature have been lacking. This study summarized and analyzed the changes of blood neural cell-derived sEVs in patients with AD for the first time. The analysis includes Aβ- and tau-related proteins, synapse related proteins, immunoinflammatory-related proteins, and miRNAs. From the cross-sectional studies, 15 proteins and genes of high diagnostic value were identified, while the longitudinal cohort studies revealed three highly predictive composite models, among which six proteins with predictive value were identified, within at least two published studies. This systematic review holds significant clinical value for the diagnosis and early prediction of AD. Furthermore, combined with other related literature, this paper compares and analyzes the results of blood neural cell-derived sEVs with other sources (CSF, brain tissue, and blood), analyzes the reasons for the differences, providing a comprehensive comparison of diagnostic biomarkers from different sources. Compared with the existing blood clinical markers, proteins in blood neural cell-derived sEVs can better reflect the pathological changes of brain tissue proteins in AD. Blood neural cell-derived sEVs not only have diagnostic value for AD, but also have early predictive value. Neurosynaptic proteins in blood neural cell-derived sEVs are newly discovered clinical diagnostic markers. Although this article shares some similarities with a systematic review published in 2021 [155], the biomarkers involved in their study were not analyzed or validated using clinical ROC curves, nor did they explore the predictive value of longitudinal studies. Furthermore, the time limit of their study was only until March 2021, but the period from 2021 to 2024 is an important period for neural cell-derived sEVs (accounting for 40%), particularly the value of synaptic proteins. Therefore, the research herein has significant advantages over previous works.

This article also has limitations. The diagnostic criteria vary (e.g., NIA-AA, NINCDS-ADRDA, IWG-2), as do the scales used (e.g., CDR, MoCA, MMSE, ADAS-cog), which may have impacted these results. In terms of the extraction method of sEVs, 33 of the 34 literatures included herein were extracted with kits, and only one study used ultracentrifugation. The results of the two extraction methods were opposite, indicating that ultracentrifugation method for the extraction was unsuitable for a small number of blood sEVs. Some studies included herein only compared between-groups differences, without conducting correlation and ROC curve analyses, thus their diagnostic value cannot be confirmed. In ROC curves, some articles only display the AUC while omitting sensitivity and specificity, which may affect the diagnostic strength of the indicator. This articles herein include 10 studies related to MCI, but only three explicitly focus on amnestic MCI (aMCI), while the remaining seven are unclear, which may introduce bias in the results regarding MCI.

Despite the tremendous potential of blood neural cell-derived sEVs, their translation into clinical practice still faces challenges. For example, the extraction and isolation methods for exosomes are not yet fully standardized, and there is a lack of strict quality control. It is essential to optimize existing extraction methods and establish standardized operational procedures to improve the quality of isolation. Emerging microfluidic technologies, for example, can enhance the sensitivity of exosome isolation by increasing the surface area between microfluidic channels and the phase of sEVs [156]. These technologies offer advantages such as simplicity, high sensitivity, and specificity [157]. Additionally, various single vesicle analysis techniques have their own strengths [158] and can significantly improve detection efficiency and accuracy, thereby enhancing the sensitivity of disease diagnosis. In future research, it would be beneficial to focus on biomarkers with high diagnostic and predictive value identified herein. Large-scale, multi-center cross-sectional studies and prospective, high-quality cohort studies should be conducted. A combination of internal validation and external validation can be used to construct a diagnostic prediction model for AD based on blood neural cell-derived sEVs, so as to provide reliable diagnostic biomarkers for early diagnosis and prediction of AD.

## CRediT authorship contribution statement

**Weibing Pan:** Writing – original draft, Visualization, Project administration, Formal analysis, Data curation, Conceptualization. **Yu Teng:** Writing – original draft, Methodology, Investigation, Formal analysis. **Xiaowan Han:** Supervision, Methodology, Funding acquisition, Data curation. **Shaojiao Liu:** Formal analysis, Data curation. **Xingxue Pang:** Data curation, Conceptualization. **Lei Wang:** Writing – review & editing, Supervision, Conceptualization. **Mingjing Zhao:** Writing – review & editing, Visualization, Project administration, Methodology, Funding acquisition, Conceptualization.

## Declaration of competing interest

The authors declare that they have no known competing financial interests or personal relationships that could have appeared to influence the work reported in this paper.

## Appendix Supplementary materials

Image, application 1

Image, application 2
