# Supplementary figures and images for: Value of blood neural cell-derived small extracellular vesicles in the diagnosis and prediction of Alzheimer's disease: A systematic review
Source: J Prev Alzheimers Dis. 2025 May 1;12(7):100193. doi: 10.1016/j.tjpad.2025.100193 (PMC12321630; doi:10.1016/j.tjpad.2025.100193)

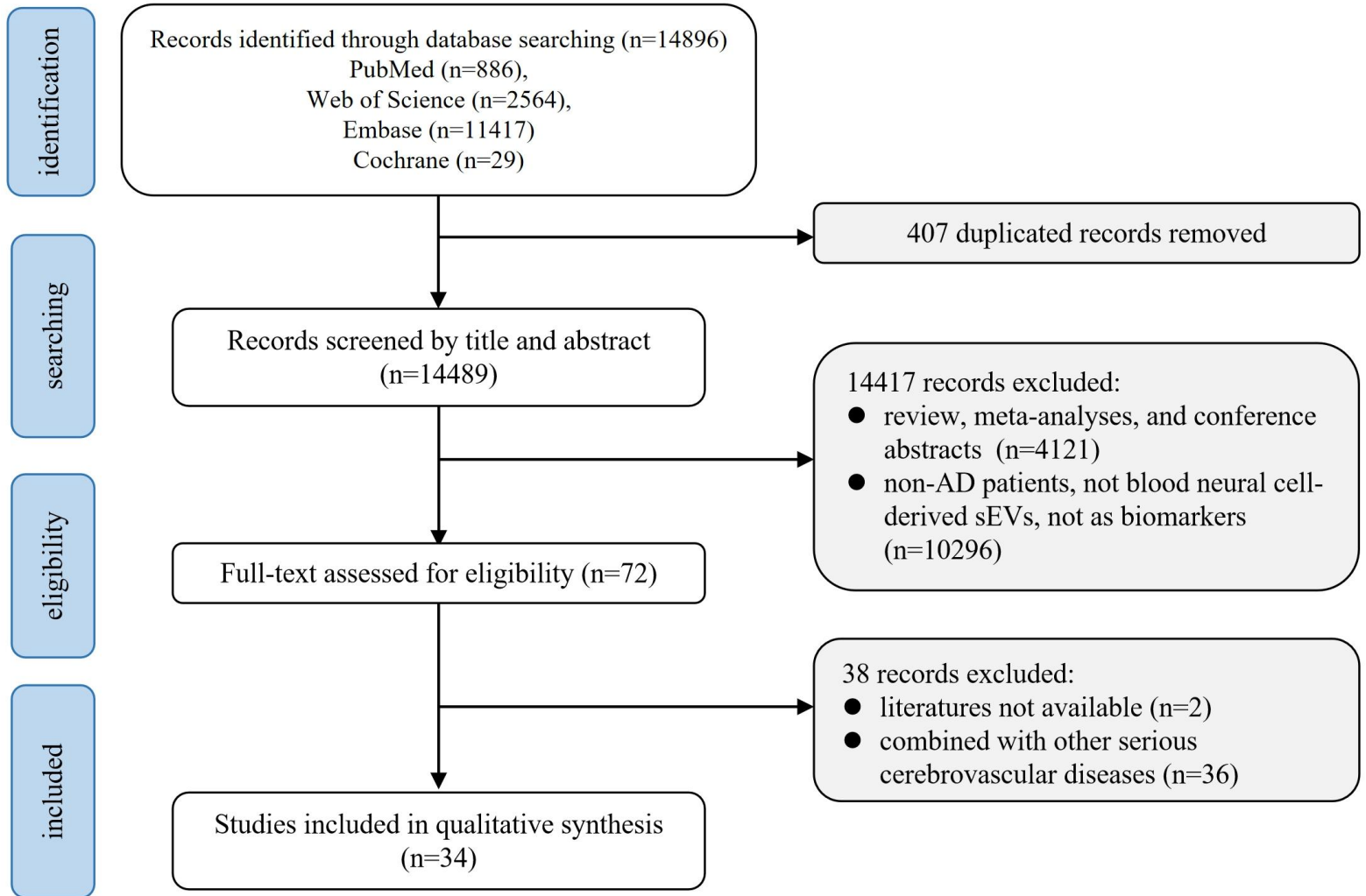

Supplement: Supplementary file 1 [file mmc1.pdf]
