# Supplementary material for: Value of blood neural cell-derived small extracellular vesicles in the diagnosis and prediction of Alzheimer's disease: A systematic review
Source: J Prev Alzheimers Dis. 2025 May 1;12(7):100193. doi: 10.1016/j.tjpad.2025.100193 (PMC12321630; doi:10.1016/j.tjpad.2025.100193)
Supplement: Supplementary file 2 [file mmc2.pdf]

Supplementary Material

Appendix 1: Search formula

Pubmed

("Alzheimer Disease"[MeSH Terms] OR ("alzheimer dementia"[Title/Abstract] OR "alzheimer dementias"[Title/Abstract] OR "dementia alzheimer"[Title/Abstract] OR "alzheimer s disease"[Title/Abstract] OR "dementia senile"[Title/Abstract] OR "senile dementia"[Title/Abstract] OR "dementia alzheimer type"[Title/Abstract] OR "alzheimer type dementia"[Title/Abstract] OR (("Alzheimer Disease"[MeSH Terms] OR ("Alzheimer"[All Fields] AND "Disease"[All Fields]) OR "Alzheimer Disease"[All Fields] OR ("Alzheimer"[All Fields] AND "Type"[All Fields] AND "Dementia"[All Fields]) OR "alzheimer type dementia"[All Fields]) AND "ATD"[Title/Abstract]) OR (("Alzheimer Disease"[MeSH Terms] OR ("Alzheimer"[All Fields] AND "Disease"[All Fields]) OR "Alzheimer Disease"[All Fields] OR ("Alzheimer"[All Fields] AND "Type"[All Fields] AND "Dementia"[All Fields]) OR "alzheimer type dementia"[All Fields]) AND "ATD"[Title/Abstract]) OR ((("Dementia"[MeSH Terms] OR "Dementia"[All Fields] OR "Dementias"[All Fields] OR "dementia s"[All Fields]) AND "Alzheimer-Type"[All Fields]) AND "ATD"[Title/Abstract]) OR "alzheimer type senile dementia"[Title/Abstract] OR "primary senile degenerative dementia"[Title/Abstract] OR "dementia primary senile degenerative"[Title/Abstract] OR "alzheimer sclerosis"[Title/Abstract] OR "sclerosis alzheimer"[Title/Abstract] OR "alzheimer syndrome"[Title/Abstract] OR "alzheimer s diseases"[Title/Abstract] OR "alzheimer diseases"[Title/Abstract] OR "alzheimers diseases"[Title/Abstract] OR "senile dementia alzheimer type"[Title/Abstract] OR "acute confusional senile dementia"[Title/Abstract] OR (("Alzheimer Disease"[MeSH Terms] OR ("Alzheimer"[All Fields] AND "Disease"[All Fields]) OR "Alzheimer Disease"[All Fields] OR ("Senile"[All Fields] AND "Dementia"[All Fields]) OR "senile dementia"[All Fields]) AND "acute confusional"[Title/Abstract]) OR "dementia presenile"[Title/Abstract] OR "presenile dementia"[Title/Abstract] OR "alzheimer disease late onset"[Title/Abstract] OR "late onset alzheimer disease"[Title/Abstract] OR (("Alzheimer Disease"[MeSH Terms] OR ("Alzheimer"[All Fields] AND "Disease"[All Fields]) OR "Alzheimer Disease"[All Fields] OR ("Alzheimer's"[All Fields] AND "Disease"[All Fields]) OR "alzheimer s disease"[All Fields]) AND "focal onset"[Title/Abstract]) OR (("Focal"[All Fields] OR "focalities"[All Fields] OR "focality"[All Fields] OR "focalization"[All Fields] OR "focalized"[All Fields] OR "focally"[All Fields] OR "focals"[All Fields] OR "local"[All Fields] OR "localisation"[All Fields] OR "localisations"[All Fields] OR "localise"[All Fields] OR "localised"[All Fields] OR "localises"[All Fields] OR "localising"[All Fields] OR "localization"[All Fields] OR "localizations"[All Fields] OR "localize"[All Fields] OR "localized"[All Fields] OR "localizer"[All Fields] OR "localizers"[All Fields] OR "localizes"[All Fields] OR "localizing"[All Fields] OR "locally"[All Fields] OR "locals"[All Fields]) AND "onset alzheimer s disease"[Title/Abstract]) OR (("Alzheimer Disease"[MeSH Terms] OR ("Alzheimer"[All Fields] AND "Disease"[All Fields]) OR "Alzheimer Disease"[All Fields] OR ("familial"[All Fields] AND "Alzheimer"[All Fields] AND "Disease"[All Fields]) OR "familial alzheimer disease"[All Fields]) AND "FAD"[Title/Abstract]) OR ((("Alzheimer Disease"[MeSH Terms] OR ("Alzheimer"[All Fields] AND "Disease"[All Fields]) OR "Alzheimer Disease"[All Fields]) AND ("familialities"[All Fields] OR "familiality"[All Fields] OR "familially"[All Fields] OR "familials"[All Fields] OR "familie"[All Fields] OR "family"[MeSH Terms] OR "family"[All Fields] OR "familial"[All Fields] OR "families"[All Fields] OR "family s"[All Fields] OR

"familys"[All Fields])) AND "FAD"[Title/Abstract]) OR ((("familialities"[All Fields] OR "familiality"[All Fields] OR "familially"[All Fields] OR "familials"[All Fields] OR "familie"[All Fields] OR "family"[MeSH Terms] OR "family"[All Fields] OR "familial"[All Fields] OR "families"[All Fields] OR "family s"[All Fields] OR "familys"[All Fields]) AND ("Alzheimer Disease"[MeSH Terms] OR ("Alzheimer"[All Fields] AND "Disease"[All Fields]) OR "Alzheimer Disease"[All Fields] OR ("Alzheimer"[All Fields] AND "Diseases"[All Fields]) OR "alzheimer diseases"[All Fields])) AND "FAD"[Title/Abstract]) OR "alzheimer disease early onset"[Title/Abstract] OR "early onset alzheimer disease"[Title/Abstract] OR "presenile alzheimer dementia"[Title/Abstract])) AND ((("Extracellular Vesicles"[MeSH Terms] OR ("extracellular vesicle"[Title/Abstract] OR "vesicle extracellular"[Title/Abstract] OR "vesicles extracellular"[Title/Abstract] OR "Exovesicles"[Title/Abstract] OR "Exovesicle"[Title/Abstract] OR "apoptotic bodies"[Title/Abstract] OR "apoptotic body"[Title/Abstract] OR "bodies apoptotic"[Title/Abstract] OR "body apoptotic"[Title/Abstract] OR "Exosome"[Title/Abstract] OR "Exosomes"[Title/Abstract])) AND ("Extracellular Vesicles"[MeSH Terms] OR ("extracellular vesicle"[Title/Abstract] OR "vesicle extracellular"[Title/Abstract] OR "vesicles extracellular"[Title/Abstract] OR "Exovesicles"[Title/Abstract] OR "Exovesicle"[Title/Abstract] OR "apoptotic bodies"[Title/Abstract] OR "apoptotic body"[Title/Abstract] OR "bodies apoptotic"[Title/Abstract] OR "body apoptotic"[Title/Abstract] OR "Exosome"[Title/Abstract] OR "Exosomes"[Title/Abstract]))) OR ("Cell-Derived Microparticles"[MeSH Terms] OR ("Cell-Derived Microparticles"[Title/Abstract] OR "cell derived microparticle"[Title/Abstract] OR (("Cell-Derived Microparticles"[MeSH Terms] OR ("Cell-Derived"[All Fields] AND "Microparticles"[All Fields]) OR "Cell-Derived Microparticles"[All Fields] OR "Microparticle"[All Fields] OR "Microparticles"[All Fields]) AND "Cell-Derived"[Title/Abstract]) OR "Ectosomes"[Title/Abstract] OR "Ectosome"[Title/Abstract] OR "microparticles cell derived"[Title/Abstract] OR "microparticles cell derived"[Title/Abstract] OR "shedding microvesicles"[Title/Abstract] OR "microvesicle shedding"[Title/Abstract] OR "microvesicles shedding"[Title/Abstract] OR "shedding microvesicle"[Title/Abstract] OR "cell membrane microparticles"[Title/Abstract] OR (("cells"[MeSH Terms] OR "cells"[All Fields] OR "Cell"[All Fields]) AND "membrane microparticle"[Title/Abstract]) OR (("membranal"[All Fields] OR "membrane s"[All Fields] OR "membraneous"[All Fields] OR "membranes"[MeSH Terms] OR "membranes"[All Fields] OR "Membrane"[All Fields] OR "membranous"[All Fields]) AND "microparticle cell"[Title/Abstract]) OR (("membranal"[All Fields] OR "membrane s"[All Fields] OR "membraneous"[All Fields] OR "membranes"[MeSH Terms] OR "membranes"[All Fields] OR "Membrane"[All Fields] OR "membranous"[All Fields]) AND "microparticles cell"[Title/Abstract]) OR (("Cell-Derived Microparticles"[MeSH Terms] OR ("Cell-Derived"[All Fields] AND "Microparticles"[All Fields]) OR "Cell-Derived Microparticles"[All Fields] OR "Microparticle"[All Fields] OR "Microparticles"[All Fields]) AND "cell membrane"[Title/Abstract]) OR "microparticles cell membrane"[Title/Abstract] OR "circulating cell derived microparticles"[Title/Abstract] OR (("Cell-Derived Microparticles"[MeSH Terms] OR ("Cell-Derived"[All Fields] AND "Microparticles"[All Fields]) OR "Cell-Derived Microparticles"[All Fields] OR ("Cell"[All Fields] AND "Derived"[All Fields] AND "Microparticle"[All Fields]) OR "cell derived microparticle"[All Fields]) AND "Circulating"[Title/Abstract]) OR ("Cell-Derived"[All Fields] AND "microparticles circulating"[Title/Abstract]) OR "circulating cell derived microparticles"[Title/Abstract] OR

"circulating cell derived microparticle"[Title/Abstract] OR (("Cell-Derived Microparticles"[MeSH Terms] OR ("Cell-Derived"[All Fields] AND "Microparticles"[All Fields]) OR "Cell-Derived Microparticles"[All Fields] OR "Microparticle"[All Fields] OR "Microparticles"[All Fields]) AND "circulating cell derived"[Title/Abstract]) OR (("Cell-Derived Microparticles"[MeSH Terms] OR ("Cell-Derived"[All Fields] AND "Microparticles"[All Fields]) OR "Cell-Derived Microparticles"[All Fields] OR "Microparticle"[All Fields] OR "Microparticles"[All Fields]) AND "circulating cell derived"[Title/Abstract]))) OR "Exosomes"[MeSH Terms])

Web of Science

(TS=(Extracellular Vesicles OR Extracellular Vesicle OR Vesicle, Extracellular OR Vesicles, Extracellular OR Exovesicles OR Exovesicle OR Apoptotic Bodies OR Apoptotic Body OR Bodies, Apoptotic OR Body, Apoptotic OR Exosome OR Exosomes) OR TS=(Cell-Derived Microparticles OR Cell Derived Microparticles OR Cell-Derived Microparticle OR Microparticle, Cell-Derived OR Ectosomes OR Ectosome OR Microparticles, Cell-Derived OR Microparticles, Cell Derived OR Shedding Microvesicles OR Microvesicle, Shedding OR Microvesicles, Shedding OR Shedding Microvesicle OR Cell Membrane Microparticles OR Cell Membrane Microparticle OR Membrane Microparticle, Cell OR Membrane Microparticles, Cell OR Microparticle, Cell Membrane OR Microparticles, Cell Membrane OR Circulating Cell-Derived Microparticles OR Cell-Derived Microparticle, Circulating OR Cell-Derived Microparticles, Circulating OR Circulating Cell Derived Microparticles OR Circulating Cell-Derived Microparticle OR Microparticle, Circulating Cell-Derived OR Microparticle, Circulating Cell-Derived) OR TS=(Cell-Derived Microparticles OR Cell Derived Microparticles OR Cell-Derived Microparticle OR Microparticle, Cell-Derived OR Ectosomes OR Ectosome OR Microparticles, Cell-Derived OR Microparticles, Cell Derived OR Shedding Microvesicles OR Microvesicle, Shedding OR Microvesicles, Shedding OR Shedding Microvesicle OR Cell Membrane Microparticles OR Cell Membrane Microparticle OR Membrane Microparticle, Cell OR Membrane Microparticles, Cell OR Microparticle, Cell Membrane OR Microparticles, Cell Membrane OR Circulating Cell-Derived Microparticles OR Cell-Derived Microparticle, Circulating OR Cell-Derived Microparticles, Circulating OR Circulating Cell Derived Microparticles OR Circulating Cell-Derived Microparticle OR Microparticle, Circulating Cell-Derived OR Microparticle, Circulating Cell-Derived)) AND TS=(Alzheimer Disease OR Alzheimer Dementia OR Alzheimer Dementias OR Dementia, Alzheimer OR Alzheimer's Disease OR Dementia, Senile OR Senile Dementia OR Dementia, Alzheimer Type OR Alzheimer Type Dementia OR Alzheimer-Type Dementia (ATD) OR Alzheimer Type Dementia (ATD) OR Dementia, Alzheimer-Type (ATD) OR Alzheimer Type Senile Dementia OR Primary Senile Degenerative Dementia OR Dementia, Primary Senile Degenerative OR Alzheimer Sclerosis OR Sclerosis, Alzheimer OR Alzheimer Syndrome OR Alzheimer's Diseases OR Alzheimer Diseases OR Alzheimers Diseases OR Senile Dementia, Alzheimer Type OR Acute Confusional Senile Dementia OR Senile Dementia, Acute Confusional OR Dementia, Presenile OR Presenile Dementia OR Alzheimer Disease, Late Onset OR Late Onset Alzheimer Disease OR Alzheimer's Disease, Focal Onset OR Focal Onset Alzheimer's Disease OR Familial Alzheimer Disease (FAD) OR Alzheimer Disease, Familial (FAD) OR Familial Alzheimer Diseases (FAD) OR Alzheimer Disease, Early Onset OR Early Onset Alzheimer Disease OR Presenile Alzheimer Dementia)

Embase

('alzheimer disease'/exp OR 'alzheimer disease' OR 'nervous disease'/exp OR 'nervous disease' OR

'nervous disorder'/exp OR 'nervous disorder' OR 'nervous system disease'/exp OR 'nervous system disease' OR 'nervous system diseases'/exp OR 'nervous system diseases' OR 'nervous system disorder'/exp OR 'nervous system disorder' OR 'neural disease'/exp OR 'neural disease' OR 'neurogenic disease'/exp OR 'neurogenic disease' OR 'neurologic complaint'/exp OR 'neurologic complaint' OR 'neurologic disorder'/exp OR 'neurologic disorder' OR 'neurologic disturbance'/exp OR 'neurologic disturbance' OR 'neurologic dysfunction'/exp OR 'neurologic dysfunction' OR 'neurologic manifestations'/exp OR 'neurologic manifestations' OR 'neurologic sign'/exp OR 'neurologic sign' OR 'neurologic symptom'/exp OR 'neurologic symptom' OR 'neurologic syndrome'/exp OR 'neurologic syndrome' OR 'neurological complaint'/exp OR 'neurological complaint' OR 'neurological deficiency'/exp OR 'neurological deficiency' OR 'neurological disease'/exp OR 'neurological disease' OR 'neurological disorder'/exp OR 'neurological disorder' OR 'neurological disturbance'/exp OR 'neurological disturbance' OR 'neurological sign'/exp OR 'neurological sign' OR 'neurological symptom'/exp OR 'neurological symptom' OR 'neurological syndrome'/exp OR 'neurological syndrome' OR 'sign, neurologic'/exp OR 'sign, neurologic' OR 'symptom, neurological'/exp OR 'symptom, neurological' OR 'neurologic disease'/exp OR 'neurologic disease') AND ('exosomes'/exp OR 'exosomes' OR 'extracellular vesicle'/exp OR 'extracellular vesicle' OR 'extracellular vesicles'/exp OR 'extracellular vesicles' OR 'exosome'/exp OR 'exosome' OR 'cell derived microparticle'/exp OR 'cell derived microparticle' OR 'cell derived microparticles'/exp OR 'cell derived microparticles' OR 'cell-derived microparticles'/exp OR 'cell-derived microparticles' OR 'circulating microparticle'/exp OR 'circulating microparticle' OR 'circulating microparticles'/exp OR 'circulating microparticles' OR 'membrane microparticles'/exp OR 'membrane microparticles' OR 'microparticle, membrane'/exp OR 'microparticle, membrane' OR 'microvesicle'/exp OR 'microvesicle' OR 'procoagulant microparticle'/exp OR 'procoagulant microparticle' OR 'procoagulant microparticles'/exp OR 'procoagulant microparticles' OR 'membrane microparticle'/exp OR 'membrane microparticle')

Cochrane

(Alzheimer Disease or Alzheimer Disease, Familial (FAD) or Familial Alzheimer Disease (FAD) or Familial Alzheimer Diseases (FAD) or Alzheimer's Disease, Focal Onset or Focal Onset Alzheimer's Disease or Acute Confusional Senile Dementia or Senile Dementia, Acute Confusional or Presenile Alzheimer Dementia or Alzheimer Disease, Early Onset or Early Onset Alzheimer Disease or Dementia, Primary Senile Degenerative or Alzheimer Type Senile Dementia or Dementia, Senile or Senile Dementia, Alzheimer Type or Dementia, Alzheimer Type or Dementia, Alzheimer or Alzheimer Sclerosis or Senile Dementia or Alzheimer Dementia or Alzheimer Dementias or Alzheimer Type Dementia or Sclerosis, Alzheimer or Alzheimer's Disease or Primary Senile Degenerative Dementia or Alzheimer Syndrome or Alzheimer-Type Dementia (ATD) or Alzheimers Diseases or Alzheimer Diseases or Alzheimer's Diseases or Alzheimer Type Dementia (ATD) or Dementia, Alzheimer-Type (ATD) or Dementia, Presenile or Presenile Dementia or Late Onset Alzheimer Disease or Alzheimer Disease, Late Onset) and (Extracellular Vesicles or Vesicles, Extracellular or Extracellular Vesicle or Vesicle, Extracellular or Exovesicles or Exovesicle or Apoptotic Body or Body, Apoptotic or Bodies, Apoptotic or Apoptotic Bodies or Exosomes or Cell-Derived Microparticles or Cell-Derived Microparticle, Circulating or Microparticle, Circulating Cell-Derived or Cell-Derived Microparticles, Circulating or Circulating Cell-Derived Microparticle or Circulating Cell-Derived Microparticles or Microparticles, Circulating Cell-Derived or Circulating Cell Derived Microparticles or

Microvesicles, Shedding or Shedding Microvesicles or Microparticle, Cell-Derived or Shedding Microvesicle or Cell Membrane Microparticle or Cell-Derived Microparticle or Ectosomes or Cell Derived Microparticles or Microparticles, Cell Derived or Microparticles, Cell-Derived or Microparticles, Cell Membrane or Membrane Microparticles, Cell or Microvesicle, Shedding or Microparticle, Cell Membrane or Ectosome or Cell Membrane Microparticles or Membrane Microparticle, Cell)

Appendix 2: quality assessment of cross-sectional studies based on the agency for healthcare research and quality (AHRQ)

**Number**	**Author/publication year**	**Q1 (1 point)**	**Q2 (1 point)**	**Q3 (1 point)**	**Q4 (1 point)**	**Q5 (1 point)**	**Q6 (1 point)**	**Q7 (1 point)**	**Q8 (1 point)**	**Q9 (1 point)**	**Q10 (1 point)**	**Q11 (1 point)**	**total scores (11 points)**	**Quality grade**
1	Ashish Kumar et al. 2023	1	1	1	1	1	1	1	1	0	1	0	9	high-quality
2	Geethu Krishna et al. 2023	1	1	0	0	1	1	0	1	0	1	0	6	moderate-quality
3	Chen Tian et al. 2022	1	1	0	1	1	1	1	1	0	1	0	8	high-quality
4	Devrim Yagmur Durur et al. 2022	1	1	0	1	1	1	0	0	0	1	0	6	moderate-quality
5	Erden Eren et al. 2022	1	0	1	0	1	1	0	0	0	1	0	5	moderate-quality
6	E. Taşdelen et al. 2022	1	1	0	1	1	1	1	1	0	1	0	8	high-quality
7	Tao-Ran Li et al. 2022	1	1	1	1	1	1	0	1	0	1	0	8	high-quality
8	X Anton Alvarez et al. 2022	1	1	1	1	1	1	1	1	0	1	0	9	high-quality
9	Ying Li et al. 2022	1	1	1	1	1	1	1	0	0	1	0	8	high-quality
10	Burak Ibrahim Arioz et al. 2021	1	1	0	1	1	1	1	1	0	1	0	8	high-quality
11	Jiacheng Zhong et al. 2021	1	1	0	1	1	1	0	1	0	1	0	7	moderate-quality
12	Longfei Jia et al. 2021	1	1	1	1	1	1	0	1	0	1	1	9	high-quality
13	Pamela J. Yao et al. 2021	1	1	0	1	1	1	0	1	0	1	0	7	moderate-quality
14	Aonan Zhao et al. 2020	1	1	1	1	1	1	1	1	0	1	1	10	high-quality
15	Dongmei Gu et al. 2020	1	1	0	1	1	1	1	1	1	1	0	9	high-quality
16	Eunjoo Nam et al. 2020	1	1	1	0	1	1	0	1	0	1	0	7	moderate-quality
17	Maria Serpente et al. 2020	1	1	1	1	1	1	0	1	0	1	0	8	high-quality
18	Nan Zhang et al. 2020	1	1	0	1	1	1	1	1	0	1	0	8	high-quality
19	Cristina Agliardi et al. 2019	1	0	0	0	1	1	0	0	0	1	0	4	moderate-quality
20	Diana J Cha et al. 2019	1	1	0	1	1	1	0	0	0	1	0	6	moderate-quality
21	Dimitrios Kapogiannis et al. 2019	1	1	0	1	1	1	0	1	0	1	1	8	high-quality
22	Longfei Jia et al. 2019	1	0	1	1	1	1	0	1	0	1	0	7	moderate-quality
23	Charisse N Winston et al. 2018	1	1	0	1	1	1	0	1	0	1	0	7	moderate-quality
24	Edward J Goetzl et al. 2018	1	1	0	1	1	1	0	1	0	1	1	8	high-quality

25	Charisse N Winston et al. 2016	1	1	0	1	1	1	1	0	0	1	0	7	moderate-quality
26	Edward J Goetzl et al. 2016	1	1	0	1	1	1	0	1	0	1	1	8	high-quality
27	Erin L. Abner et al. 2016	1	0	0	1	1	1	0	0	0	1	1	6	moderate-quality
28	Dimitrios Kapogiannis et al. 2015	1	1	0	1	1	1	0	1	0	1	1	8	high-quality
29	Edward J Goetzl et al. 2015	1	1	0	1	1	1	0	1	0	1	1	8	high-quality
30	Edward J. Goetzl et al. 2015	1	1	0	1	1	1	0	1	0	1	1	8	high-quality
31	Massimo S. Fiandaca et al. 2015	1	1	0	1	1	1	0	1	0	1	1	8	high-quality
32	Charisse N. Winston et al. 2019	1	1	0	1	1	1	0	1	0	1	0	7	moderate-quality
33	Edward J. Goetzl et al. 2017	1	1	0	1	1	1	0	1	0	1	1	8	high-quality
34	Edward J Goetzl et al. 2016	1	1	0	1	1	1	0	1	0	1	0	7	moderate-quality

The total score is 11 points, with 0–3 considered low-quality, 4–7 deemed moderate-quality, and 8–11 classified as high quality.

Q1: Define the source of information (survey, record review). Q2: List inclusion and exclusion criteria for exposed and unexposed subjects (cases and controls) or refer to previous publications. Q3: Indicate time period used for identifying patients. Q4: Indicate whether or not subjects were consecutive if not population-based. Q5: Indicate if evaluators of subjective components of study were masked to other aspects of the status of the participants. Q6: Describe any assessments undertaken for quality assurance purposes (e.g., test/retest of primary outcome measurements). Q7: Explain any patient exclusions from analysis. Q8: Describe how confounding was assessed and/or controlled. Q9: If applicable, explain how missing data were handled in the analysis. Q10: Summarize patient response rates and completeness of data collection. Q11: Clarify what follow-up, if any, was expected and the percentage of patients for which incomplete data or follow-up was obtained.

Appendix 3: quality assessment of cohort studies based on the newcastle-ottawa scale (NOS)

Number	Author/publication year	Q1 (1 point)	Q2 (1 point)	Q3 (1 point)	Q4 (1 point)	Q5 (2 points)	Q6 (1 point)	Q7 (1 point)	Q8 (1 point)	total scores (9 points)	Quality grade
1	Longfei Jia et al. 2021. Alzheimers Dement	1	0	1	1	2	1	1	0	7	high-quality
2	Aonan Zhao et al. 2020. Transl Neurodegener	1	1	1	1	2	1	1	0	8	high-quality
3	Dimitrios Kapogiannis et al. 2019. JAMA Neurol	1	0	1	1	0	1	1	0	5	high-quality
4	Edward J Goetzl et al. 2018. FASEB J	0	1	1	1	1	1	1	0	6	high-quality
5	Edward J Goetzl et al. 2016. FASEB J	1	1	1	1	1	1	0	0	6	high-quality
6	Erin L. Abner et al. 2016. Ann Clin Transl Neurol	1	1	0	1	1	1	1	0	6	high-quality
7	Dimitrios Kapogiannis et al. 2015. FASEB J	1	1	1	1	1	1	1	0	7	high-quality
8	Edward J Goetzl et al. 2015. Neurology	1	1	1	1	1	1	1	0	7	high-quality
9	Edward J. Goetzl et al. 2015. Ann Clin Transl Neurol	1	1	0	1	1	1	1	0	6	high-quality
10	Massimo S. Fiandaca et al. 2015. Alzheimers Dement	1	1	0	1	1	1	1	0	6	high-quality
11	Edward J. Goetzl et al. 2017. Ann Neurol	1	1	0	1	2	1	1	0	7	high-quality

The total score is 9 points, with 0–4 considered low-quality, 5–9 deemed high quality.
Q1: Is the case definition adequate? Q2: Representativeness of the cases. Q3: Selection of Controls. Q4: Definition of Controls. Q5: Comparability of cases and controls on the basis of the design or analysis. Q6: Ascertainment of exposure. Q7: Same method of ascertainment for cases and controls. Q8: Non-Response rate.

Appendix 4: changes of proteins or genes from different sources (blood neural cell-derived sEVs, brain tissue, blood, CSF) in AD

Classification of sEV molecules	molecules	blood neural cell-derived sEVs	brain tissue	blood	CSF
Aβ- and tau- related proteins	Aβ42/40	↑	↑ (mouse)	↓ (plasma)	↓
	Aβ42	↑	↑ (human)<br>↑ (rat)	↓ (plasma)	↓
	p-Tau181	↑	↑ (rat)	↑ (plasma)	↑
	p-S396-Tau	↑	↑ (mouse)	↑ (plasma)	
Synaptic proteins	neurogranin	↓	↓	no change	↑
	synaptotagmin	↓	↓		↑
	synaptopodin	↓	↓		
	synaptophysin	↓	↓ (human)<br>↓ (mouse)		
	NMDAR2A	↓	↓ (rat)		
miRNAs	miR-29a-5p	↑	↓	↓ (serum)	↑
	miR-29c-3p	↑	↓	↓ (serum)	↓
	miR-106b-5p	↑	↓	↑ (serum)<br>↓ (serum)	
	miR-107	↑	↓ (human)<br>↓ (mouse)		
	miR-125b-5p	↑	↑	↓ (plasma)<br>↓ (serum)<br>↑ (serum)	↑, ↓
	miR-132-5p	↑	↓	↑ (serum)	
	let-7e-5p	↑	↓	↓ (blood)<br>↑ (plasma)<br>↓ (plasma)	

↑: increased; ↓: decreased; Arrows (not marked with source) means they are from humans.
AD: Alzheimer's disease; CSF: cerebrospinal fluid; NMDAR2A: N-methyl-D-aspartate receptor 2A.
